# Mineral-driven pyrolysis chemistry and temperature-dependent pyrolysis of textile wastewater sludge: kinetics, reaction pathways, and char functionality

**DOI:** 10.1039/d6ra07938e

**Published:** 2026-09-01

**Authors:** Gaurav Singh, Ranjeet Kumar Mishra, Neeraj Kumar

**Affiliations:** a Department of Applied Sciences, J. B. Institute of Technology, Veer Madho Singh Bhandari Uttarakhand Technical University Dehradun- 248015 India vatsneeraj78@gmail.com; b Manipal Institute of Technology, Manipal Academy of Higher Education Manipal India ranjeet.mishra@manipal.edu

## Abstract

This study investigated the pyrolysis behaviour, conversion-dependent kinetics, and char production potential of textile wastewater sludge (TXT) for sustainable waste valorisation. The feedstock contained 45.84 wt% volatile matter, 24.09 wt% fixed carbon, 20.25 wt% ash, and exhibited a significantly higher heating value (HHV) of 11.98 MJ kg^−1^. Thermogravimetric analysis at 10, 20, and 30 °C min^−1^ identified three major degradation stages, with increasing heating rate shifting decomposition towards higher temperatures. The kinetic analysis over *α* = 0.1–0.8 using KAS, OFW, DAEM, Tang, and VZ methods yielded average activation energies of 213.03, 218.61, 208.81, 157.72, and 170.14 kJ mol^−1^, confirming the multistep and conversion-dependent nature of TXT decomposition. Criado master-plot analysis indicated predominantly diffusion-controlled behaviour at lower conversion, with increasing contributions from reaction-order and nucleation-related mechanisms at higher conversion. Char production at 450–800 °C reduced the yield from 51.20 to 43.53 wt% while generally increasing aromaticity, alkalinity, and pore development. Among the tested temperatures, char produced at 700 °C showed the most favourable properties, with 44.40 wt% carbon, HHV of 18.21 MJ kg^−1^, a BET surface area of 55.23 m^2^ g^−1^, and a water-holding capacity of 64.82 wt%. These findings demonstrate the potential of pyrolysis for converting TXT into a stable, mineral-rich carbonaceous material with relevance to environmental remediation, adsorption, and resource recovery.

## Abstract 2

This study investigated the pyrolysis behaviour, conversion-dependent kinetics, and char production potential of textile wastewater sludge (TXT) for sustainable waste valorisation.

## Graphical abstract

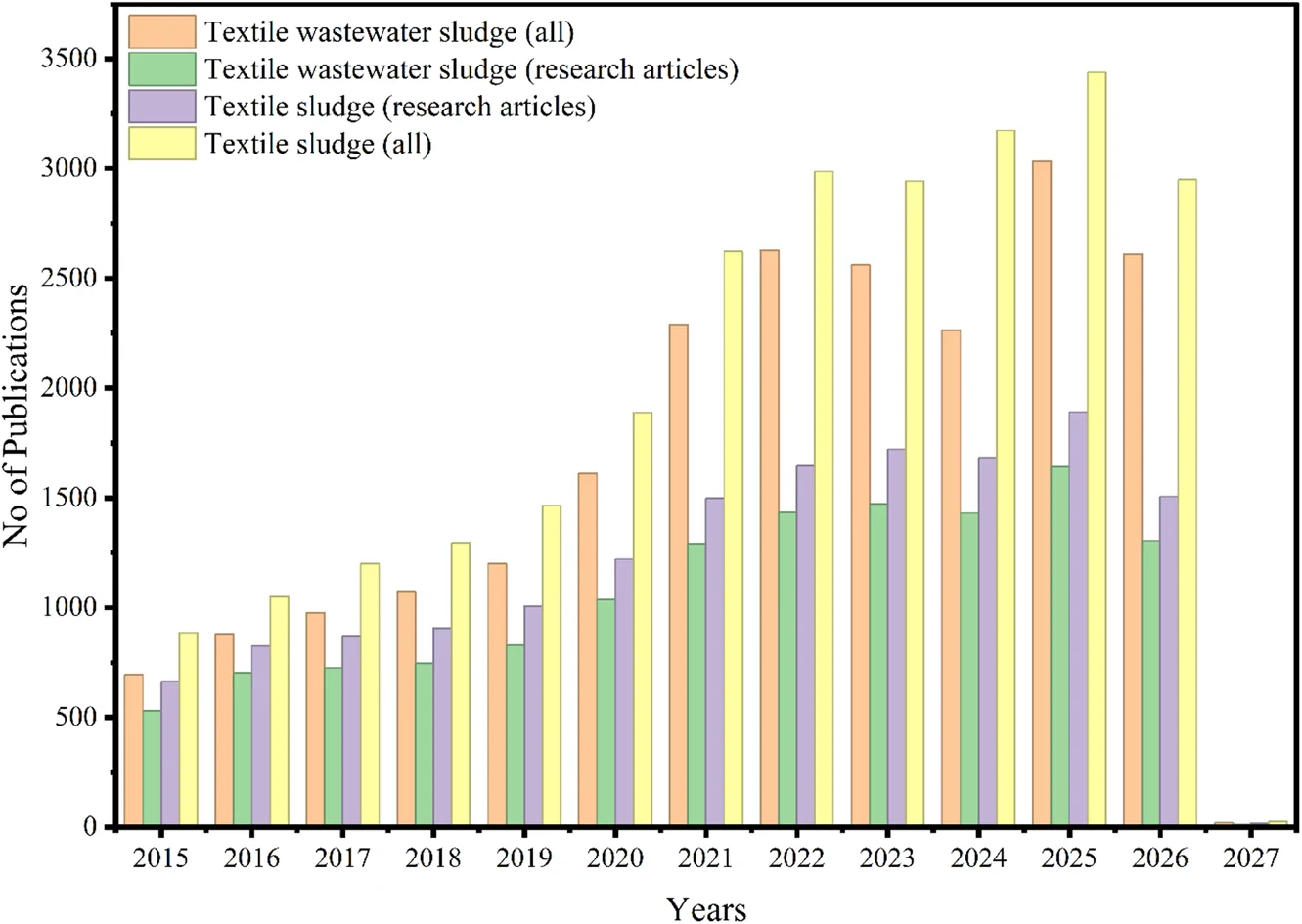

## 1 Introduction

The textile manufacturing industry is one of the major water and chemical-intensive industrial sectors, generating substantial quantities of wastewater during processes such as desizing, scouring, bleaching, dyeing, printing and finishing. These wastewater streams contain suspended solids, residual dyes, organic compounds, salts, surfactants and other inorganic constituents. Worldwide textile consumption has reached 7–13 kg per capita, contributing to an estimated 134 million tonnes of textile waste by 2030. Nevertheless, only 15–16% of the annual textile waste generated is recycled.^1^ Certain textile materials are highly resistant to degradation and may persist in landfill environments for more than 200 years, exacerbating long-term environmental impacts.^2^ Treatment of textile wastewater generates considerable quantities of sludge, commonly referred to as textile wastewater sludge or textile dyeing sludge, which poses a significant waste-management challenge due to its heterogeneous organic and inorganic composition. Improper disposal can lead to contaminant leaching, soil and water pollution, odour generation, and long-term waste-management challenges, requiring sustainable treatment and resource-recovery strategies. Conventional management of textile wastewater sludge, including landfilling and other disposal-based approaches, can result in the loss of potentially recoverable organic and mineral resources and raise concerns about contaminant leaching and long-term environmental stability.^3^ Conventional disposal practices may occupy valuable land and pose risks of contaminant leaching and long-term environmental management challenges.^4^ Technologies capable of simultaneously reducing sludge volume and recovering value from its organic fraction are required.

Pyrolysis has attracted increasing attention for the valorisation of industrial sludges because it can convert the organic fraction into gaseous and condensable products while retaining a substantial fraction of carbon and inorganic minerals in the solid char.^5^ Char is a porous, carbon-rich material with high surface area and abundant surface functional groups, making it promising for environmental remediation and pollutant adsorption. Its porous structure and surface chemistry can also provide favourable sites for microbial immobilization and enhance the stability and functionality of biochar-based systems.^6,7^ The properties and potential applications of the resulting char depend strongly on the composition of the feedstock and the pyrolysis temperature.^8^ The thermal decomposition of textile wastewater sludge is considerably more complex than that of relatively homogeneous lignocellulosic biomass because of the coexistence of organic matter, polymers, dyes, salts and mineral constituents. Understanding its conversion behaviour over a range of temperatures and heating rates is essential for process design and optimisation. Thermogravimetric analysis (TGA) combined with iso-conversional kinetic methods can provide information on the apparent activation energy and conversion-dependent reaction behaviour without assuming a single reaction mechanism.^9^ Kinetic evaluation is necessary to understand the energy barriers associated with different stages of conversion. Non-isothermal model-free approaches are useful for complex feedstocks because they permit activation energy to be evaluated as a function of conversion without assuming a single reaction mechanism.^10^ The use of multiple kinetic methods can improve interpretation because individual approaches employ different mathematical approximations.^11^ Kissinger–Akahira–Sunose (KAS), Ozawa–Flynn–Wall (OFW), Distributed Activation Energy Model (DAEM), Tang (TG), and Vyazovkin (VZ) were used for the kinetic study. These methods were selected because they are widely accepted iso-conversional kinetic models that provide reliable estimates of activation energy without assuming a reaction mechanism. Thermodynamic parameters and Criado master plots are employed to elucidate energetic and mechanistic characteristics.^12^ Kinetic analysis combined with thermodynamic parameters such as enthalpy change (Δ*H*), Gibbs free energy change (Δ*G*), and entropy change (Δ*S*) provides deeper insight into the energetic requirements and feasibility of thermal decomposition.^11^

Several studies have demonstrated the potential of pyrolysing wastewater sludge to produce value-added products. Wahab *et al.* (2022) investigated thermal degradation kinetics and catalytic pyrolysis of sludge from textile and leather industrial wastewater. Results showed that TGA identified three stages: dehydration, volatile degradation, and carbonaceous decomposition, with major DTG peaks at 305–332 and 444–465 °C. Non-catalytic activation energies were found to be 182.77, 170.63, and 175.83 kJ mol^−1^ using KAS, FWO, and Friedman methods. The use of catalysts reduced the activation energy. Y-zeolite increased the oil HHV to 34.1 MJ kg^−1^, while Al_2_O_3_ improved oil quality by deoxygenating and reducing fatty acids.^13^ The study did not demonstrate any char production and its applications. Wang *et al.* (2024) investigated the synergetic co-pyrolysis of waste textiles with Ca-rich and Fe-rich industrial sludge. Co-pyrolysis increased total weight loss to 69.81% and 70.01%, compared with 50.05% and 49.13% for individual sludges.^7^ Further, the activation energy decreased by approximately 50%, with the three-dimensional diffusion (D3) model providing the best fit. Co-pyrolysis lowered CO_2_ evolution temperatures by 32–85 °C, increased *C* < 10 compounds to 93% and 88%, and enhanced aldehyde, ketone, and stable char formation.^13^ This study did not focus on the char production and its application.^4^ Zhang *et al.* (2017) demonstrated that continuous microwave pyrolysis effectively converts textile dyeing sludge into value-added char. Char yield decreased to 63.87 wt% at 750 °C, while condensate yield peaked at 650 °C. The maximum BET surface area reached 91.90 m^2^ g^−1^ at 550 °C, sludge volume was reduced by 60–66%, and heavy-metal leaching remained below regulatory limits, confirming the environmental safety of the produced char.^14^ Tang *et al.* (2022) demonstrated that co-pyrolysis of textile dyeing sludge with waste tyres significantly enhances thermal conversion. The TP55 blend achieved the highest weight loss (68.92%) and CPI (7.44 × 10^−6^% min^−2^ K^−3^), whereas TR55 recorded a weight loss of 55.30%.^15^ Co-pyrolysis lowered activation energy, reduced CO, SO_2_, and nitrogen-containing emissions, and improved hydrocarbon-rich oil and aromatic char production through strong synergistic interactions.^15^ Cao *et al.* (2022) investigated textile dyeing sludge pyrolysis, revealing that the primary decomposition occurs at 127–557 °C with activation energy increasing from 64.70 to 90.20 kJ mol^−1^ following the Avrami–Erofeev (*n* = 3) model. 10 wt% CaO at 650 °C achieved >70% sulphur retention, significantly reducing H_2_S emissions and improving environmentally sustainable sludge valorization.^16^

Despite the increasing interest in the pyrolysis of TXT, few studies have systematically evaluated its conversion-dependent thermal degradation behaviour and the temperature-dependent properties of the resulting char.^17^ The variation of apparent activation energy with conversion and the corresponding changes in the apparent solid–state reaction behaviour require further investigation for this heterogeneous feedstock. The influence of pyrolysis temperature on the yield, char characteristics, calorific value, surface area, and water-holding capacity of char derived from textile wastewater sludge has not been comprehensively assessed in a single study. Based on these considerations, it was hypothesised that the heterogeneous organic and mineral composition of textile wastewater sludge would result in multistep, conversion-dependent thermal degradation, with the apparent activation energy and dominant reaction behaviour varying with conversion. It was hypothesised that increasing pyrolysis temperature would progressively enhance carbonisation, mineral enrichment, surface development, calorific value, and water-holding capacity of the resulting char, although excessive thermal severity could reduce char yield. Therefore, an optimum pyrolysis temperature was expected to provide a favourable balance between char yield and its physicochemical properties.

**Fig. 1:**
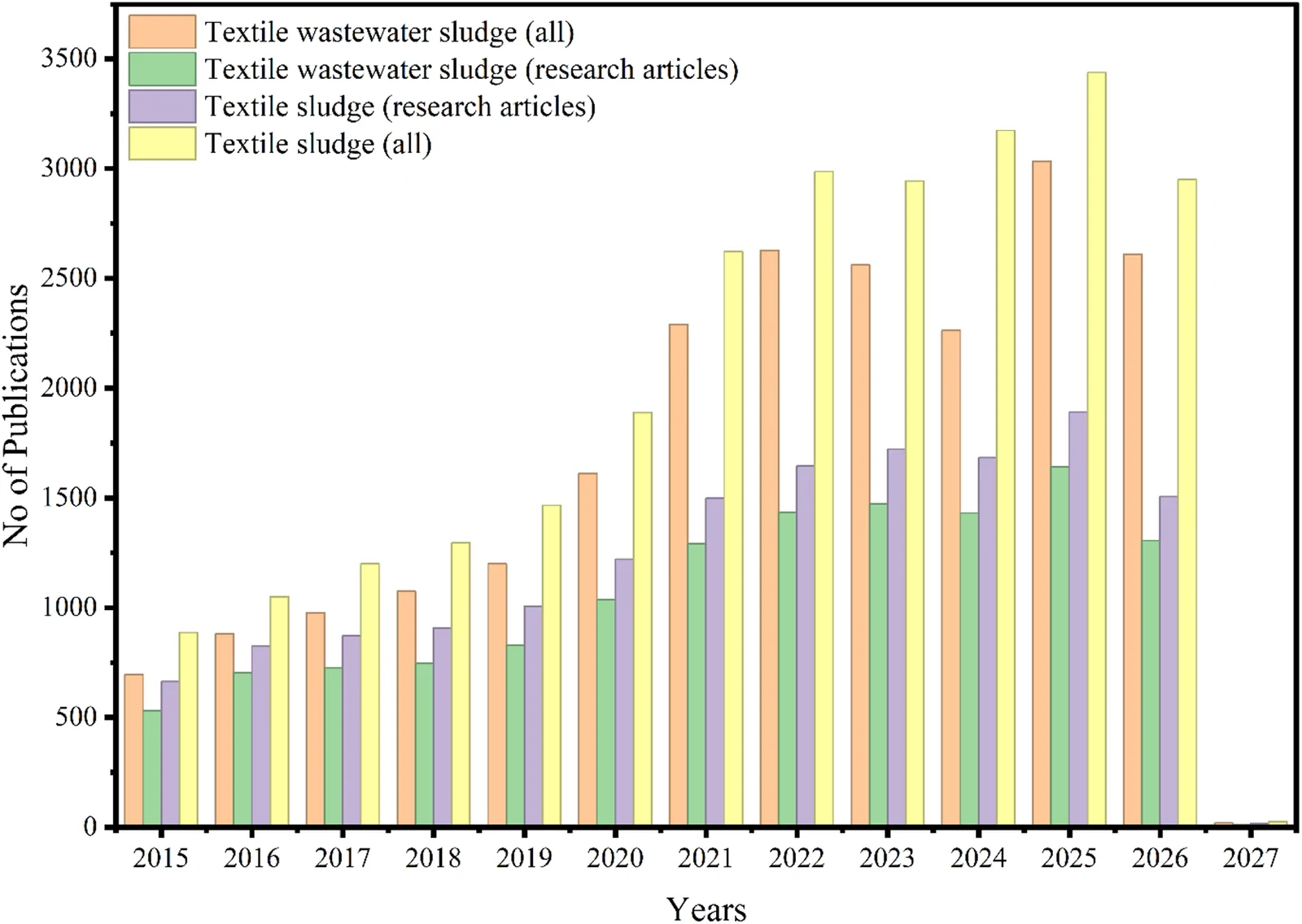
Scopus publication trends for textile wastewater sludge and textile sludge from 2015 to 2027.

The present work investigates the thermal decomposition of textile wastewater sludge at different heating rates (10, 20, and 30 °C min^−1^) using multiple iso-conversional kinetic approaches (KAS, OFW, DAEM, TG, and VZ methods) and evaluates the reaction behaviour using the Criado master-plot method. Char produced at different pyrolysis temperatures (450, 600, 700, and 800 °C) is subsequently characterised to determine the temperature-dependent changes in its physicochemical and functional properties. The objective is to provide a systematic understanding of the thermal conversion behaviour of textile wastewater sludge and the resulting evolution of char properties. The increasing scientific interest in textile sludge and textile wastewater sludge is reflected in the Scopus publication trends shown in Fig. 1. The literature output shows a clear and substantial increase in research interest over the study period (except year 2027). Publications containing the term “textile sludge” increased from approximately 900 records in 2015 to nearly 3400 records in 2025, while “textile wastewater sludge” increased from approximately 700 to more than 3000 records during the same period. Textile articles followed a similar upward trend, reaching approximately 1900 articles for “textile sludge” and 1650 articles for “textile wastewater sludge” in 2025. The 2026 records already indicate substantial research activity despite representing only part of the year.

## 2 Materials and methods

This section describes the materials, experimental procedures, analytical techniques, and theoretical approaches employed to investigate the pyrolysis behaviour of textile wastewater sludge (TXT). The methodology includes sample collection and preparation, physicochemical characterisation, thermogravimetric and FTIR analyses, kinetic and thermodynamic evaluations, Criado master-plot analysis, and laboratory-scale pyrolysis for char production and characterisation.

### 2.1. Sample collection and preparation

Textile wastewater sludge (TXT) was collected from the Muzaffarnagar district, Uttar Pradesh, India. The collected sludge was initially sun-dried for 1 week to reduce its moisture content, then oven-dried at 70 °C for 48 h to ensure uniform moisture removal. The dried sample was then passed through a magnetic separator to remove any metal impurities. The glass fragments and other foreign materials were manually removed to obtain a clean feedstock. The processed TXT was stored in an airtight glass container to prevent moisture absorption prior to further characterisation and pyrolysis experiments.

### 2.2. Physicochemical characterisation of feedstock

The proximate and elemental analyses were conducted to evaluate the suitability of the feedstock for pyrolysis. Proximate analysis was performed in accordance with the ASTM standard (Table 1). The moisture content (MC) was determined by drying approximately 1.0 g of sun-dried biomass in a hot-air oven (Optics Technology, Delhi, India) at 105 °C for 1 h. The dried sample was cooled in a desiccator to room temperature, and the moisture content was calculated from the difference between the initial and final masses. Volatile matter (VM) was determined by heating 1.0 g of the oven-dried sample in a covered ceramic crucible at 925 ± 10 °C for 7 min in a muffle furnace (Optics Technology, Delhi, India). After cooling in a desiccator, the mass loss was recorded as the volatile matter content. Ash content was measured by heating 1.0 g of the oven-dried biomass at 575 ± 10 °C for 3 h in the same muffle furnace. The sample was subsequently cooled in a desiccator, reheated until a constant mass was achieved, and the remaining residue was reported as the ash content. Fixed carbon (FC) was calculated by difference. Elemental composition (C, H, and O) was used to determine the CHO index of textile waste sludge using eqn (1), thereby assessing its chemical characteristics and suitability for thermochemical conversion. Ultimate analysis of TXT was conducted using a CHNS/O elemental analyser (Thermo Fisher Scientific, Flash Smart V CHNS/O, IIT Bombay) to determine its elemental composition. Approximately 3.0 mg of each sample was weighed into tin capsules and analysed under standard operating conditions. The analyser was equipped with dual reactive beds consisting of copper oxide and electrolytic copper, maintained at 900 °C, and coupled with a thermal conductivity detector (TCD). Argon gas (99.90% purity) was used as the carrier gas at a flow rate of 140 mL min^−1^, while oxygen was supplied as the reactive gas at a flow rate of 250 mL min^−1^. The weight percentages of carbon (C), hydrogen (H), and nitrogen (N) were directly determined, whereas the oxygen (O) content was calculated by difference (without ash content). The HHV of the TXT was determined using an oxygen bomb calorimeter (Parr 6400). The bulk density (BD), which affects feedstock storage, transportation, handling, reactor feeding, and thermochemical conversion performance, was determined by measuring the mass of biomass occupying a known volume with a graduated cylinder and a digital analytical balance.1
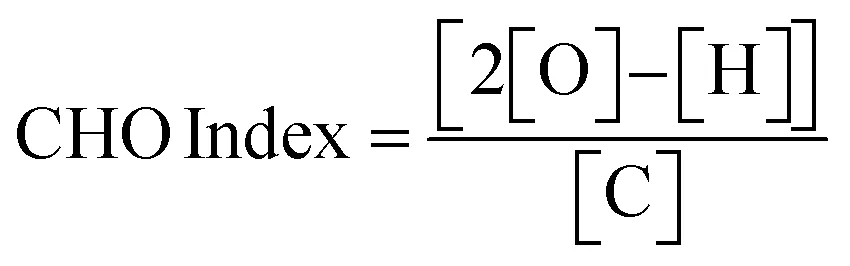


**Table 1:** Test standards and experimental procedures used for proximate analysis of textile waste sludge

Property (wt%)	Test standard	Apparatus	Procedures
Moisture content	ASTM E1756	Rotek hot-air oven (250 °C), Mumbai, India	The sample was dried at 105 °C for at least 1 h
Volatile matter	ASTM E872	Ants Innovations-2171 Pvt. Ltd, Mumbai, India	The sample was heated to 925 °C and held at that temperature for 7 min
Ash content	ASTM E1755	Ants Innovations-2171 Pvt. Ltd, Mumbai, India	The sample was heated to 575 °C for 3 h
Fixed carbon	—	By difference	It is calculated by subtracting the combined contents of moisture, ash, and volatile matter from 100 wt%

The CHO index was calculated from the molar fractions of carbon (C), hydrogen (H), and oxygen (O) using eqn (1). The CHO index typically ranges from −4 to +4, where negative values indicate a lower degree of oxidation and higher fuel quality, whereas positive values indicate a greater degree of oxidation, generally associated with lower energy content. The CHO index serves as a useful indicator of the fuel characteristics and the higher heating value (HHV) of the feedstock. The inorganic elemental composition of the biomass was determined by inductively coupled plasma-optical emission spectroscopy (ICP-OES; PerkinElmer Optima 5300DV) after acid digestion. Silica (SiO_2_) content was quantified spectrophotometrically from the ash residue according to the method described by Bridgeman *et al.* (2007).^12^ The ash chemistry indices, including the base-to-acid ratio (B/A), alkali index (AI), iron-to-calcium ratio (Fe/Ca), and total alkali (TA), were calculated using eqn (2)–(5) to assess the ash behaviour and predict potential slagging, fouling, and corrosion tendencies during thermochemical conversion.2
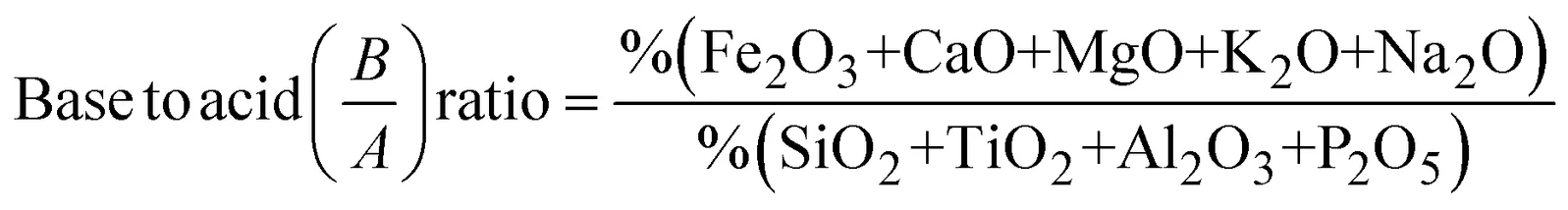
3
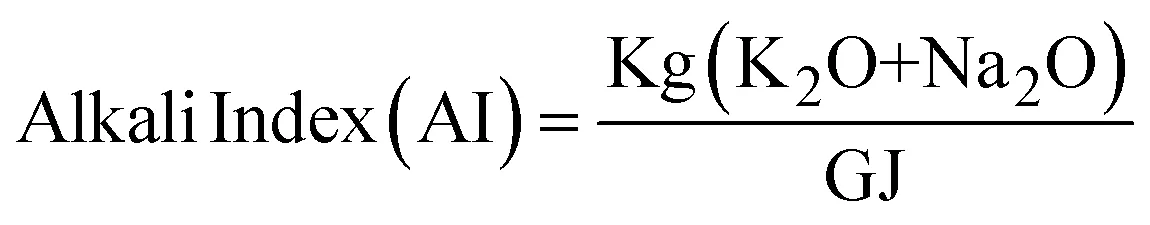
4
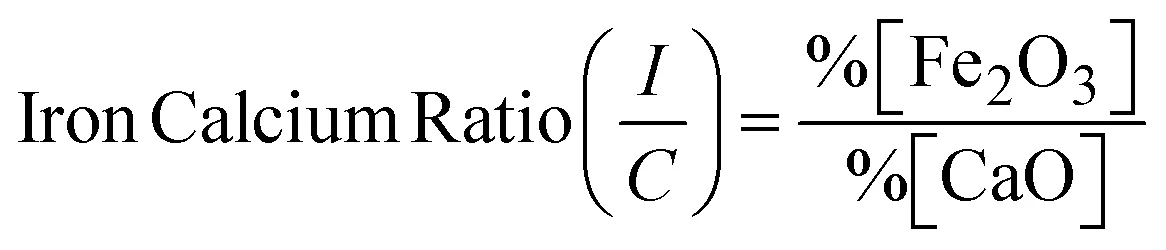

5 Total Alkalis (TA) = % Na_2_O + % K_2_O

### 2.3. Thermal and FTIR analysis of TXT

The thermal degradation behaviour and pyrolysis characteristics of TXT were investigated using a thermogravimetric analyser (Hitachi TA-7000) under an inert nitrogen atmosphere. Approximately 8.0 mg of oven-dried biomass was placed in an alumina (Al_2_O_3_) crucible and heated from 30 to 900 °C at a heating rate of 10 °C min^−1^ under a continuous nitrogen flow of 50 mL min^−1^ to evaluate its thermal stability. To investigate the effect of heating rate on pyrolysis behaviour and determine the kinetic parameters, thermogravimetric experiments were performed at heating rates of 10, 20 and 30 °C min^−1^ under identical experimental conditions. The surface functional groups of the samples were characterised using Fourier transform infrared spectroscopy (FTIR; Bruker Alpha II). Prior to analysis, the samples were oven-dried at 105 °C for 1 h and finely ground. The dried biomass was thoroughly mixed with spectroscopic-grade KBr (99.9% purity, analytical grade) at a 1 : 100 mass ratio to obtain well-defined absorption spectra. FTIR spectra were recorded over the wavenumber range of 400–4000 cm^−1^ with a spectral resolution of 4 cm^−1^ and a scan acquisition time of 40 s. The spectra obtained were used to identify the major functional groups associated with the organic and inorganic constituents of TXT.

### 2.4. Kinetic theory

TXT is a heterogeneous feedstock containing organic polymers, proteins, dyes, synthetic fibres, and inorganic mineral constituents; its thermal decomposition involves numerous overlapping and consecutive reactions. The pyrolysis process involves a series of overlapping and interconnected reactions, making it difficult to establish a single, definitive reaction mechanism. During pyrolysis, thousands of parallel and consecutive reactions, including dehydration, depolymerisation, decarboxylation, decarbonylation, cracking, cyclisation, aromatisation, radical recombination, and secondary vapour-phase reactions, occur within a short residence time, resulting in the simultaneous formation of char, condensable vapours (pyrolysis oil), and gases. The relative contribution of these reactions is strongly influenced by feedstock composition, heating rate, temperature, residence time, and the presence of catalysts. Owing to the highly complex nature of these reaction networks, elucidating the complete reaction mechanism remains challenging. A generalised reaction pathway is proposed to illustrate the thermal decomposition routes and the principal transformations leading to gaseous, liquid, and solid products. Table 2 includes the various model-free methods used in the present study.

6 Biomass (solid) → Vollatiles (gas) + solid (biochar/char)

**Table 2:** Adopted kinetics and thermodynamic models for the calculation of kinetic parameters

Model Name	Equations
Kissinger–Akahira–Sunose (KAS)	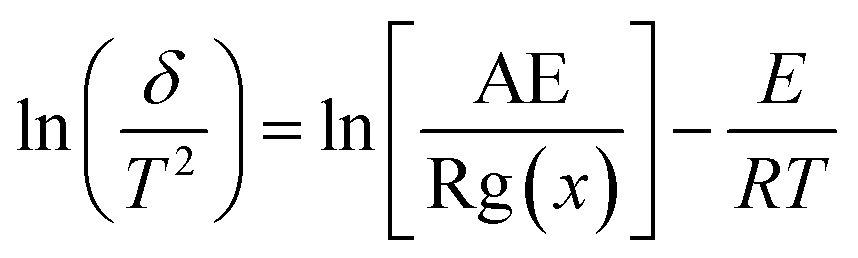 (7)
Ozawa–Flynn–Wall (OFW)	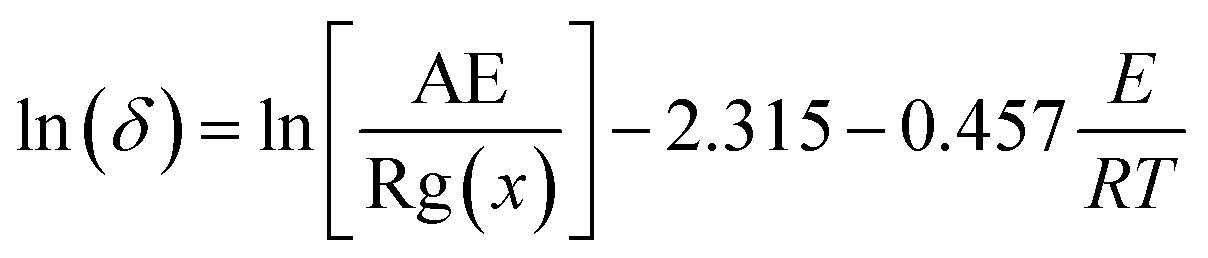 (8)
Tang Method (TG)	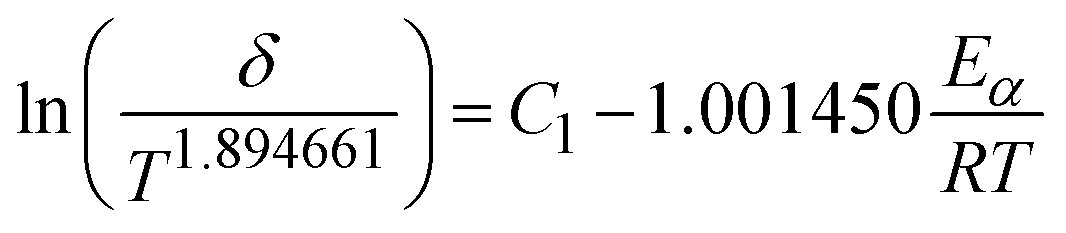 (9)
Distributed Activated Energy Model (DAEM)	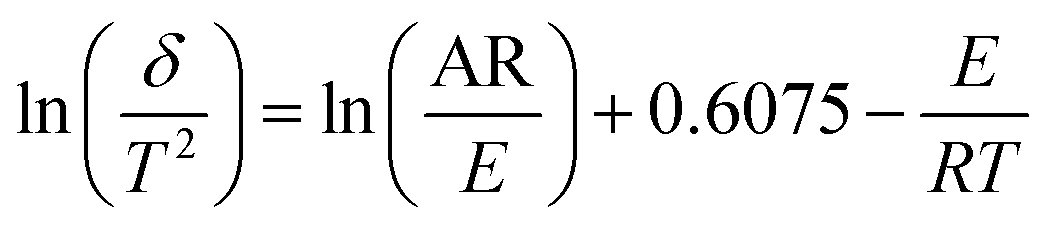 (10)
Vyazovkin Model (VZ)	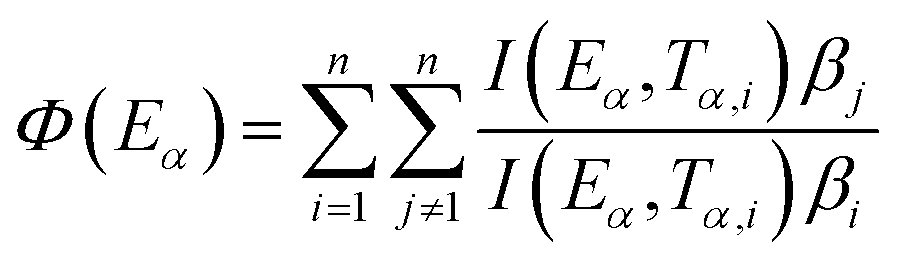 (11)
	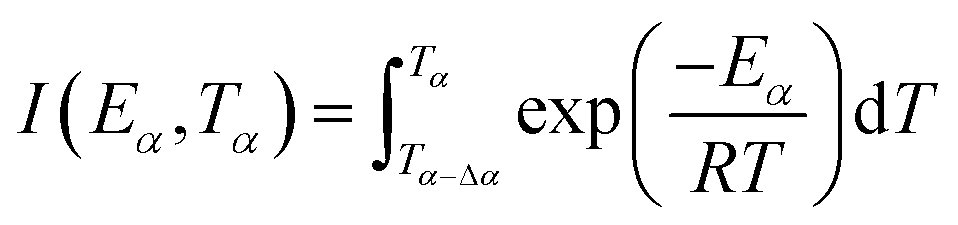 (12)

**Thermodynamic analysis**	
Enthalpy	Δ*H* =*E* − *RT*_m_ (13)
Gibbs free energy	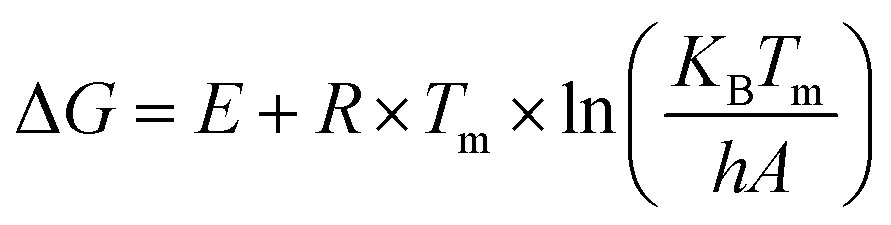 (14)
Entropy	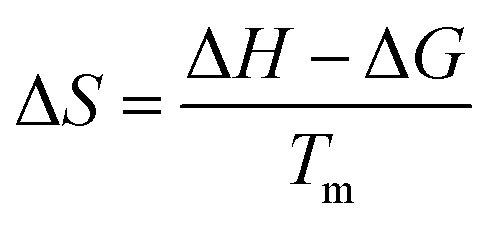 (15)

### 2.5. Master plot (Criado method)

XT is a complex heterogeneous matrix containing organic and inorganic constituents; its thermal degradation during pyrolysis involves numerous simultaneous and consecutive reactions, making the decomposition mechanism highly complex. The solid–state reaction mechanism can be investigated using the Criado master plot method, which provides a reliable approach for identifying the most probable reaction model by comparing experimental and theoretical master plots (Criado *et al.*, 1989). The Criado method can be expressed as:16
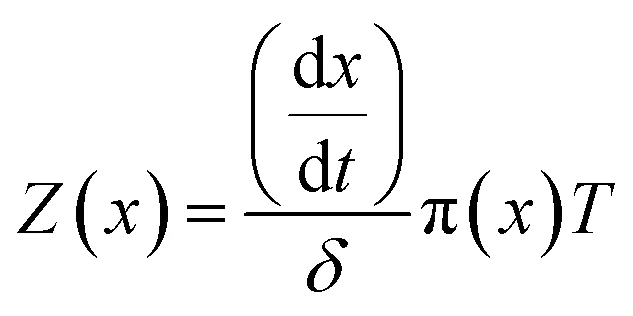
where 
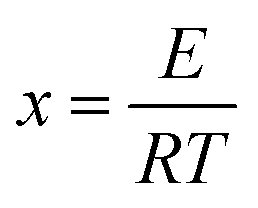
 and π (*x*) is an approximation of the temperature integral. The approximation proposed by Senum and Yang was used to solve the equation, yielding negligible error (<10^−5^ for *x* > 20). The master curves corresponding to the different reaction models listed in Table 3 were generated as a function of conversion using eqn (16).

17 *Z*(*x*) = *f*(*x*) × *g*(*x*)

**Table 3:** Algebraic expressions for *f*(*α*) and *g*(*α*) for the solid–state reaction models considered in the Criado master-plot analysis ^17,10^

Reaction mechanisms	Symbols	*f*(*α*)	*g*(*α*)
**Reaction order**			
First order	*F*_1_	(1 − *α*)	−ln(1 − *α*)
Second order	*F*_2_	(1 − *α*)^2^	(1 − *α*)^−1^ − 1
Third order	*F*_3_	(1 − *α*)^3^	[(1 − *α*)^−2^ − 1]/2

**Diffusion**			
One-way transport	*D*_1_	0.5*α*	*α* ^2^
Two-way transport	*D*_2_	[−ln(1 − *α*)]^−1^	*α* + (1 − *α*)ln(1 − *α*)
Three-way transport	*D*_3_	1.5(1 − *α*)^2/3^[1 − (1 − *α*)^1/3^]^−1^	[1 − (1 − *α*)^1/3^]^2^
Ginstling–Brounshtein equation	*D*_4_	1.5[(1 − *α*)^1/3^ − 1]^−1^	(1 − 2*α*/3) − (1 − *α*)^2/3^

**Limiting the surface reaction between both phases**			
One dimension	*R*_1_	1	*α*
Two-dimension	*R*_2_	2(1 − *α*)^1/2^	1 − (1 − *α*)^1/2^
Three-dimension	*R*_3_	3(1 − *α*)^2/3^	1 − (1 − *α*)^1/3^

**Random nucleation and nuclei growth**			
Two dimensional	*A*_2_	2(1 − *α*)[−ln(1 − *α*)]^1/2^	[−ln(1 − *α*)]^1/2^
Three dimensional	*A*_3_	3(1 − *α*)[−ln(1 − α)]^2/3^	[−ln(1 − *α*)]^1/3^

**Exponential nucleation**			
Power law, *n* = 1/2	*P*_2_	2*α*^1/2^	*α* ^1/2^
Power law, *n* = 1/3	*P*_3_	3*α*^2/3^	*α* ^1/3^
Power law, *n* = 1/4	*P*_4_	4*α*^3/4^	*α* ^1/4^

Rearranging eqn (16) and (17) give,18
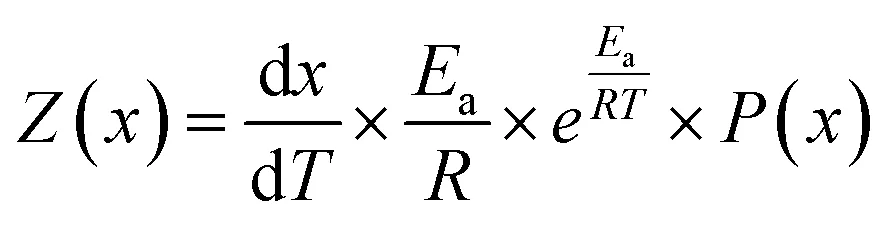


Eqn (18) was used to construct the experimental master curves. The reaction mechanism governing thermal decomposition was identified by comparing the experimental and theoretical master curves. The various reaction mechanisms considered for biomass pyrolysis are summarised in Table 3.

### 2.6. Char production

The pyrolysis experiments were carried out in a laboratory-scale fixed-bed reactor under an inert nitrogen (N_2_) atmosphere. The setup is made of stainless steel (SS301) with a diameter of 15 cm and a length of 30 cm. The experimental setup consisted of an N_2_ cylinder, a flow-control valve, a fixed-bed pyrolysis reactor placed inside an electrically heated furnace, a water-cooled condenser, and a bio-oil collection flask. Before each experiment, nitrogen gas was introduced into the reactor at a controlled flow rate (100 mL min^−1^) to purge air and establish an oxygen-free environment. A known quantity of dried TXT (100 g) was loaded into the reactor and heated to the desired pyrolysis temperature. During heating, the biomass underwent thermal decomposition, producing solid char and volatile pyrolysis products. The char remained inside the reactor, while the volatile vapours were continuously carried by the nitrogen stream toward the condensation system. The char was produced at 450, 600, 700, and 800 °C with a heating rate of 10 °C min^−1^ and a holding time of 45 min. The vapours were passed through a water-cooled condenser, where the condensable fraction was cooled and collected as a liquid in a collection flask. The remaining non-condensable gases were discharged through the gas outlet. After completion of the experiment, the reactor was allowed to cool under continuous nitrogen flow before the char was recovered and weighed. eqn (19) is used to calculate the char yield. The systematic presentation of the pyrolysis experimental setup is listed in Fig. 2.19
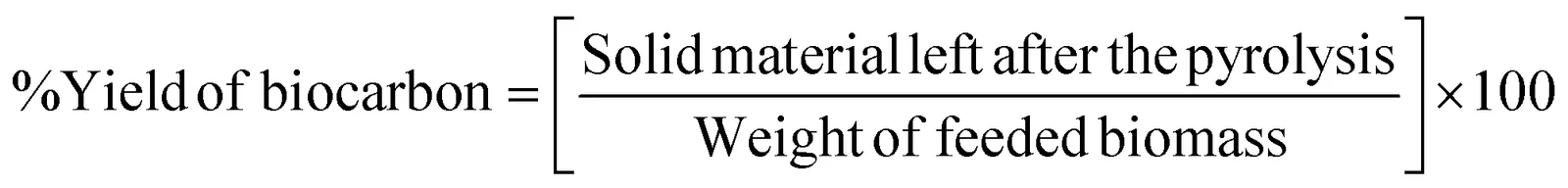


**Fig. 2:**
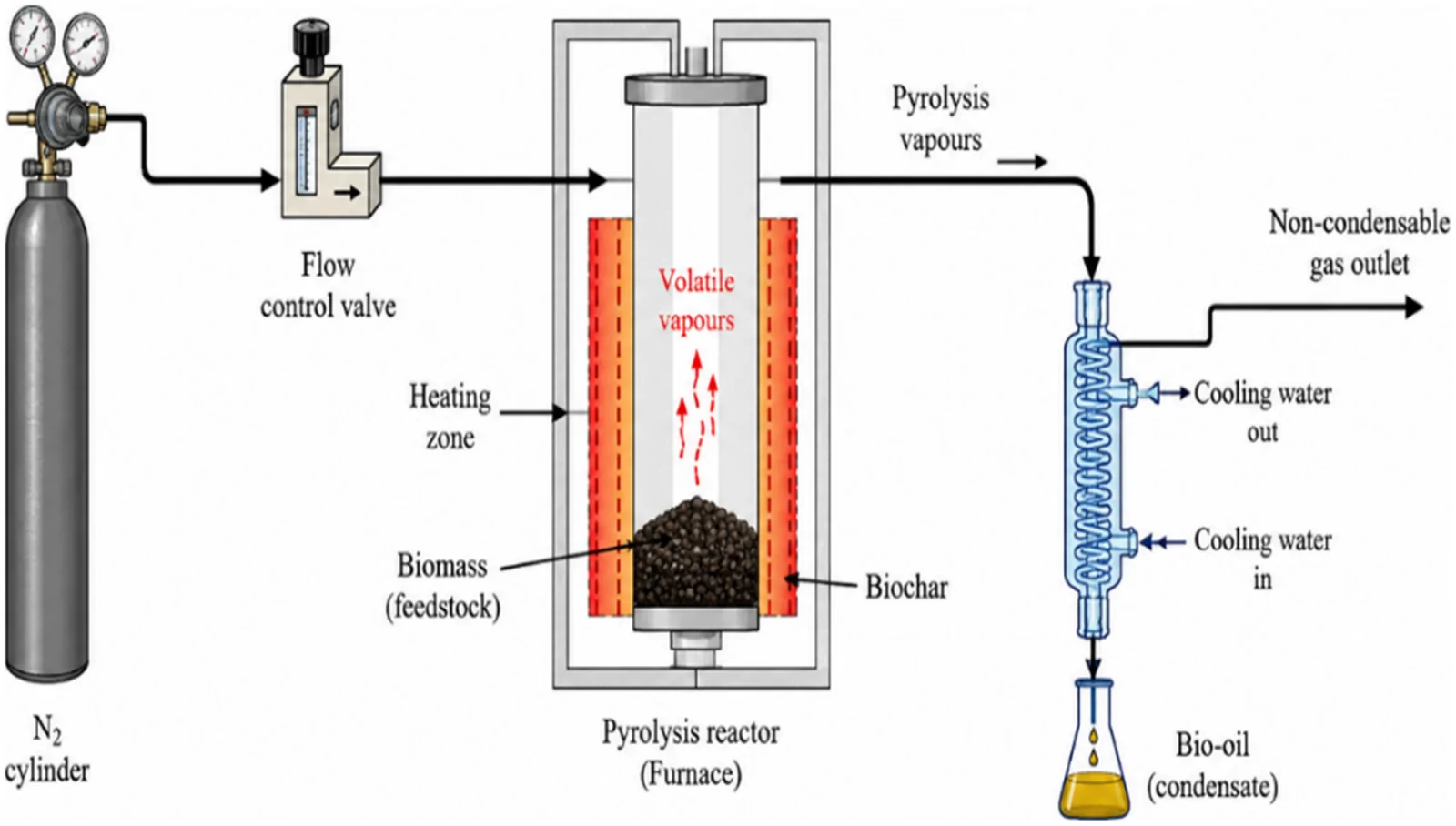
The schematic representation of the experimental pyrolysis setup to produce char.

### 2.7. Characterization of char

The proximate analysis (moisture and ash content), elemental analysis, higher heating value (HHV), and bulk density of the char samples were determined as described in Section 2.2. The specific surface area was measured using an Autosorb-iQ surface area analyser (Quantachrome Instruments, USA). The char samples were degassed at 300 °C for 180 min to remove physically adsorbed moisture and volatile contaminants. Nitrogen adsorption–desorption isotherms were obtained, and the Brunauer–Emmett–Teller (BET) surface area was calculated in accordance with ASTM D6556-19. The surface morphology of the char was characterized using a Scanning Electron Microscope (FESEM; Zeiss Supra 40, Germany) coupled with an Energy Dispersive X-ray Spectrometer (EDS; Oxford Inca Energy 350, UK). The oven-dried char samples were mounted on carbon tape and sputter-coated with a thin double layer of gold to minimise charging effects. Micrographs were acquired under high-vacuum conditions at accelerating voltages of 10 kV. The pH of the char was determined by dispersing 1 g of oven-dried char in 10 mL of deionised water. The pH meter was calibrated using standard buffer solutions of pH 4.0, 7.0, and 10.0. The water-holding capacity (WHC) was determined using a ceramic Büchner funnel fitted with Fisherbrand P8 filter paper and deionised water, following the standard gravimetric filtration method.

### 2.8. Experimental reproducibility and statistical analysis

All physicochemical characterisation (excluding TGA and FTIR, SEM) and char property measurements were performed in triplicate (*n* = 3), and the results are reported as mean ± standard deviation (SD). The standard deviation was calculated from independent experimental measurements to assess experimental variability and reproducibility. Kinetic parameters were calculated from thermogravimetric analysis performed at three heating rates (10, 20, and 30 °C min^−1^). The reproducibility of the kinetic calculations was evaluated by assessing the consistency of the conversion-dependent activation energies and the corresponding coefficients of determination (*R*^2^) from the individual kinetic models.

## 3 Results and discussions

This section presents and discusses the experimental results from the physicochemical characterisation, thermal degradation, and kinetic and thermodynamic analyses of textile wastewater sludge, as well as the temperature-dependent properties and structural characteristics of the resulting char. The results are discussed in relation to the feedstock composition, pyrolysis behaviour, reaction mechanisms, and the potential valorisation of the char produced.

### 3.1. Physicochemical characterization of TXT

The physicochemical characterisation of TXT is presented in Table 4 and compared with other biomass, such as Textile dyeing sludge,^18^ Sewage Sludge,^19^ Textile wastewater sludge (TWS),^20^ and Textile dyeing sludge (DS).^21^ The results revealed that the TXT possessed a moisture content of 9.82 ± 1.44 wt%, volatile matter of 45.84 ± 0.31 wt%, ash content of 20.25 ± 0.53 wt%, fixed carbon of 24.09 wt%, and a higher heating value (HHV) of 11.98 ± 0.23 MJ kg^−1^. These characteristics indicate that the investigated sludge contains a considerable proportion of combustible organic matter while maintaining a relatively low inorganic fraction, making it a promising feedstock for pyrolysis and other waste-to-energy technologies.^22^ The MC of the present TXT sample was below 10 wt%, indicating that the sludge had undergone effective dewatering prior to characterisation. Moisture is one of the most critical parameters affecting thermal conversion, as a significant portion of the supplied heat is consumed in water evaporation before decomposition begins. Lower moisture content improves overall pyrolysis energy efficiency by reducing drying requirements and increasing the effective energy available for thermal degradation.^22^ Compared with previously reported textile dyeing sludge (39 wt%), textile wastewater sludge (6.50 wt%), and sewage sludge (7 wt%), the present TXT sample exhibited a comparatively low moisture content. The present sample falls within the desirable range for thermochemical processing, requiring only minimal pretreatment before conversion.^23^ The volatile matter of TXT was found to be 45.84 wt%, confirming that the sludge contains a substantial quantity of thermally degradable organic constituents. During pyrolysis, volatile compounds decompose rapidly, producing condensable vapours and permanent gases that directly influence the production of pyrolysis oil and syngas. The relatively high volatile matter content indicates that TXT contains a substantial fraction of thermally degradable material that may contribute to condensable and gaseous products during pyrolysis.^24^ Compared with previously published TDS, DS, and SS, the volatile matter of the present sample is considerably higher, indicating a greater abundance of degradable organic compounds. This difference is likely attributable to variations in textile manufacturing processes, wastewater treatment technologies, dye compositions, chemical additives, and sludge stabilisation methods employed across different industrial facilities. Such variations significantly influence the organic composition of sludge and its thermal behaviour.^24^

**Table 4:** Characterisation of TXT and comparison with published studies

Analysis	Textile wastewater sludge (TXT)	Textile dyeing sludge^18^	Sewage sludge (SS)^19^	Textile wastewater sludge (TWS)^20^	Textile dyeing sludge (DS)^21^
**Proximate analysis (wt%)**					
Moisture content	9.82 ± 1.44	39.18	7.30	6.50	—
Volatile matter	45.84 ± 0.311	23.66	38	53.20	23.90
Ash content	20.25 ± 0.53	56.98	46.80	33.60	59.38
Fixed carbon	24.09	19.36	7.90	6.70	16.72

**Ultimate analysis (wt%)**					
Carbon	38.75 ± 0.16	30.54 ± 0.34	28	28.81	26.52
Hydrogen	4.43 ± 0.13	2.20 ± 0.09	3.4	4.94	2.30
Oxygen	55.00 ± 0.18	7.74 ± 0.63	10.30	60.39	9.02
Nitrogen	1.20 ± 0.11	1.44 ± 0.07	4.0	4.83	1.30
Sulphur	0.61	0.79 ± 0.28	0.90	1.03	1.10
H/C	1.37	—	—	—	—
O/C	1.06	—	—	—	—
CHO index	0.75	—	—	—	—
HHV (MJ kg^−1^)	11.98 ± 0.23	9.51 ± 0.17	10.90	11.60	9.02
Bulk density (kg m^−3^)	405.64 ± 2.27	—		492	

The ash content of the investigated TXT was 20.25 wt%, lower than the values reported for several comparative sludge samples listed in Table 4. A lower ash content is advantageous because ash represents non-combustible mineral matter that reduces the overall fuel quality and limits the formation of volatile products during pyrolysis. Excessive ash may also lead to operational challenges, including slagging, fouling, and catalyst deactivation during thermal conversion.^25^ The relatively lower ash content indicates a greater proportion of combustible organic matter and may be favourable for thermochemical conversion. The fixed carbon content of TXT was 24.09 wt%, higher than that of the biomass reported in Table 4, indicating a greater tendency towards char formation during pyrolysis. Higher fixed carbon generally results in greater char production, improved carbon stability, and enhanced structural integrity.^25^

Ultimate analysis of TXT supports the favourable fuel characteristics. The carbon content (38.75 wt%) was notably higher than that reported in most studies in Table 4, suggesting a greater proportion of energy-rich organic compounds. Carbon is the principal contributor to fuel value, and its higher concentration generally enhances thermal conversion efficiency and calorific value.^24^ Similarly, the hydrogen content (4.43 wt%) contributes positively to the material's energy density and favours hydrocarbon formation during pyrolysis.^24^ The relatively high oxygen content (55 wt%) indicates the presence of oxygen-containing organic and inorganic constituents and may favour the formation of oxygenated pyrolysis products (Table 4). High oxygen content generally promotes the formation of oxygenated compounds, including acids, alcohols, aldehydes, ketones, and phenolic derivatives, during pyrolysis.^24^ The nitrogen (1.20 wt%) and sulphur (0.61 wt%) contents of TXT were lower than those reported in the study (Table 4), which is environmentally beneficial because these elements are precursors to nitrogen oxides (NO_*x*_) and sulphur oxides (SO_*x*_) during combustion or gasification. The lower concentrations reduce harmful emissions and minimise the need for expensive gas-cleaning systems, which improves the environmental sustainability of thermochemical conversion.^24^

The H/C ratio (1.37) indicates moderate hydrogen content and limited aromaticity, while the O/C ratio (1.06) reflects abundant oxygen-containing functional groups, resulting in moderate fuel quality. Lower H/C and O/C ratios after pyrolysis signify increased carbonization, calorific value, improved thermal stability, and enhanced char quality. During pyrolysis, progressive dehydration, decarboxylation, and decarbonylation reactions are expected to decrease these atomic ratios, leading to increased carbonisation and formation of highly aromatic char. Such structural evolution generally enhances the thermal stability, surface chemistry, and adsorption performance of the resulting char.^26,27^ The calculated CHO index of TXT was 0.75, indicating a moderate degree of oxidation and the presence of substantial oxygen-containing constituents.^28^ This result is consistent with the high oxygen content (55.00 wt%) and the moderate HHV of 11.98 MJ kg^−1^ reported for TXT. The positive CHO index suggests comparatively lower fuel quality than highly reduced carbonaceous feedstocks. Nevertheless, the value indicates that TXT remains suitable for thermochemical conversion, where deoxygenation and carbonisation can improve fuel properties. The higher heating value (HHV) of TXT (11.98 MJ kg^−1^) exceeded the value presented in Table 4, confirming its superior energy recovery potential. Although the calorific value remains lower than that of conventional lignocellulosic biomass due to its relatively high oxygen content, it is still sufficient to support efficient thermochemical conversion.^27^ The bulk density of TXT was 405.64 kg m^−3^, indicating favourable storage and handling characteristics for continuous reactor feeding. The combined proximate and ultimate analyses of TXT indicate that the investigated textile sludge possesses desirable fuel properties, including low moisture content, moderate volatile matter, relatively low ash content, high fixed carbon, elevated carbon concentration, and improved calorific value.

### 3.2. Burnout temperature

**Fig. 3:**
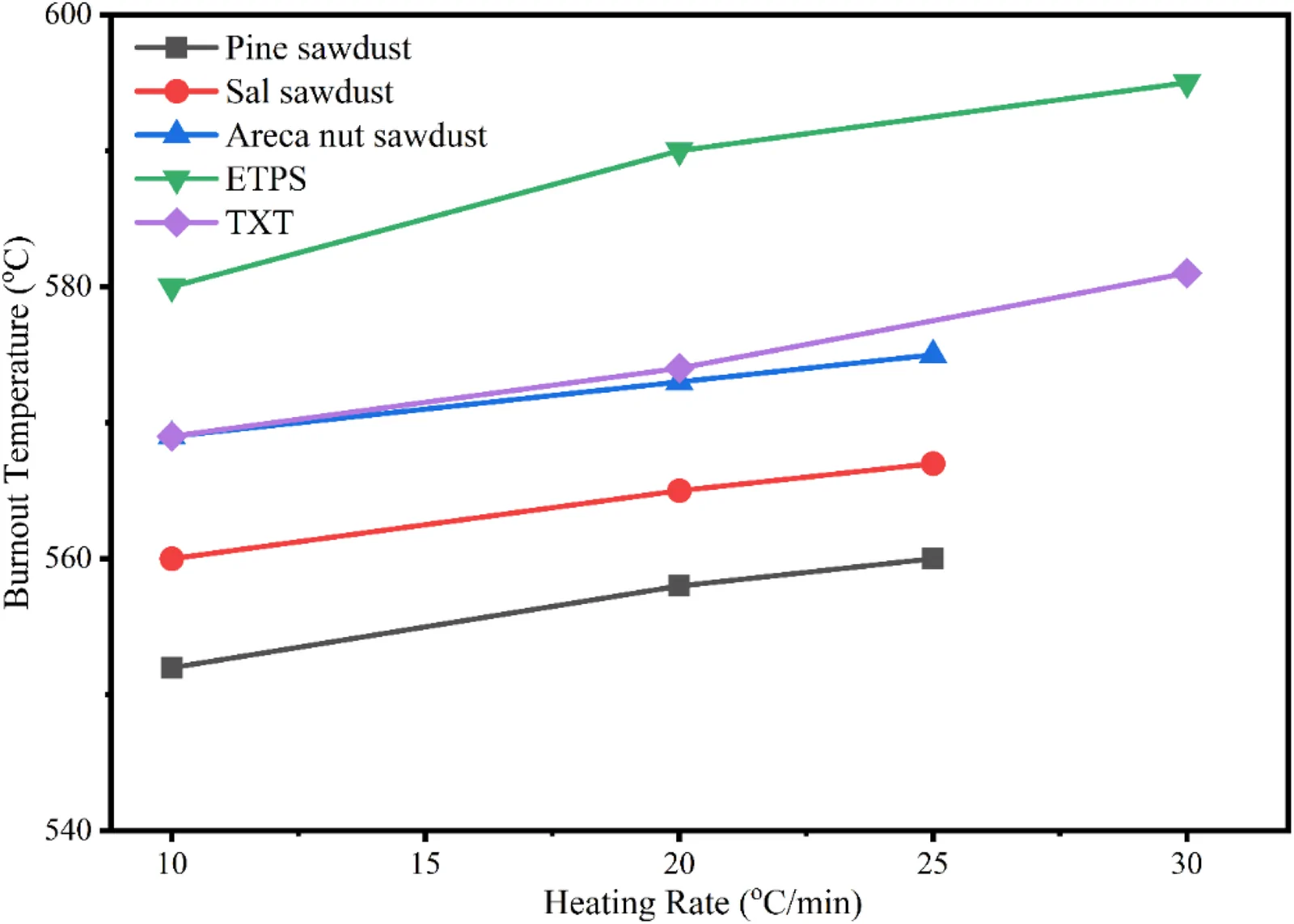
Burnout temperature of TXT compared with other reported feedstocks.

The burnout temperature profiles illustrate that heating rate affects the thermal decomposition behaviour of different waste feedstocks, providing valuable insights into their pyrolysis reactivity and thermal stability. The burnout temperature is the temperature at which nearly all combustible material has been consumed, leaving primarily ash. It is a useful comparative parameter for evaluating the thermal-degradation behaviour and apparent reactivity of different feedstocks. The burnout temperature is influenced by several factors, including feedstock composition, heating rate, residence time, and the presence of catalysts. The burnout temperatures of the ETPS,^29^ TXT, Areca nut sawdust, Sal sawdust and Pine sawdust^30^ are listed in Fig. 3. TXT exhibited a gradual increase in burnout temperature from approximately 569 °C at 10 °C min^−1^ to 581 °C at 30 °C min^−1^. This trend indicates reduced pyrolysis reactivity at higher heating rates due to thermal lag.^29^ The relatively high burnout temperature suggests moderate thermal stability, likely resulting from its higher ash and mineral content. The burnout temperature of all investigated feedstocks increased with increasing heating rate, indicating that rapid heating delayed the completion of thermal degradation. This behaviour can be attributed to thermal lag and reduced residence time, which require higher temperatures to achieve complete conversion.^31^ Among the tested feedstocks, ETPS exhibited the highest burnout temperature, increasing from approximately 580 °C at 10 °C min^−1^ to 595 °C at 30 °C min^−1^, reflecting its lower reactivity and greater thermal stability. TXT and areca nut sawdust showed intermediate burnout temperatures of about 581 °C and 575 °C, respectively, at the highest heating rate. In contrast, sal sawdust and pine sawdust exhibited lower burnout temperatures (567 °C and 560 °C at 25 °C min^−1^), suggesting relatively higher pyrolysis reactivity and easier thermal decomposition. Burnout temperature is widely used to evaluate and compare the thermal degradation and pyrolysis behaviour of biomass. Mishra *et al.* (2024) demonstrated that burnout temperature is strongly influenced by the heating rate. They reported that waste nitrile gloves exhibited the lowest burnout temperature, decreasing from approximately 610 °C at 10 °C min^−1^ to 520 °C at 50 °C min^−1^, owing to their high volatile matter and polymeric composition, which promote rapid thermal decomposition at higher heating rates.^32^ In contrast, neem seeds showed relatively higher burnout temperatures, declining only slightly from 605 °C to 560 °C, indicating comparatively greater thermal stability due to their oil-rich composition.^32^

### 3.3. Effect of heating rates

**Fig. 4:**
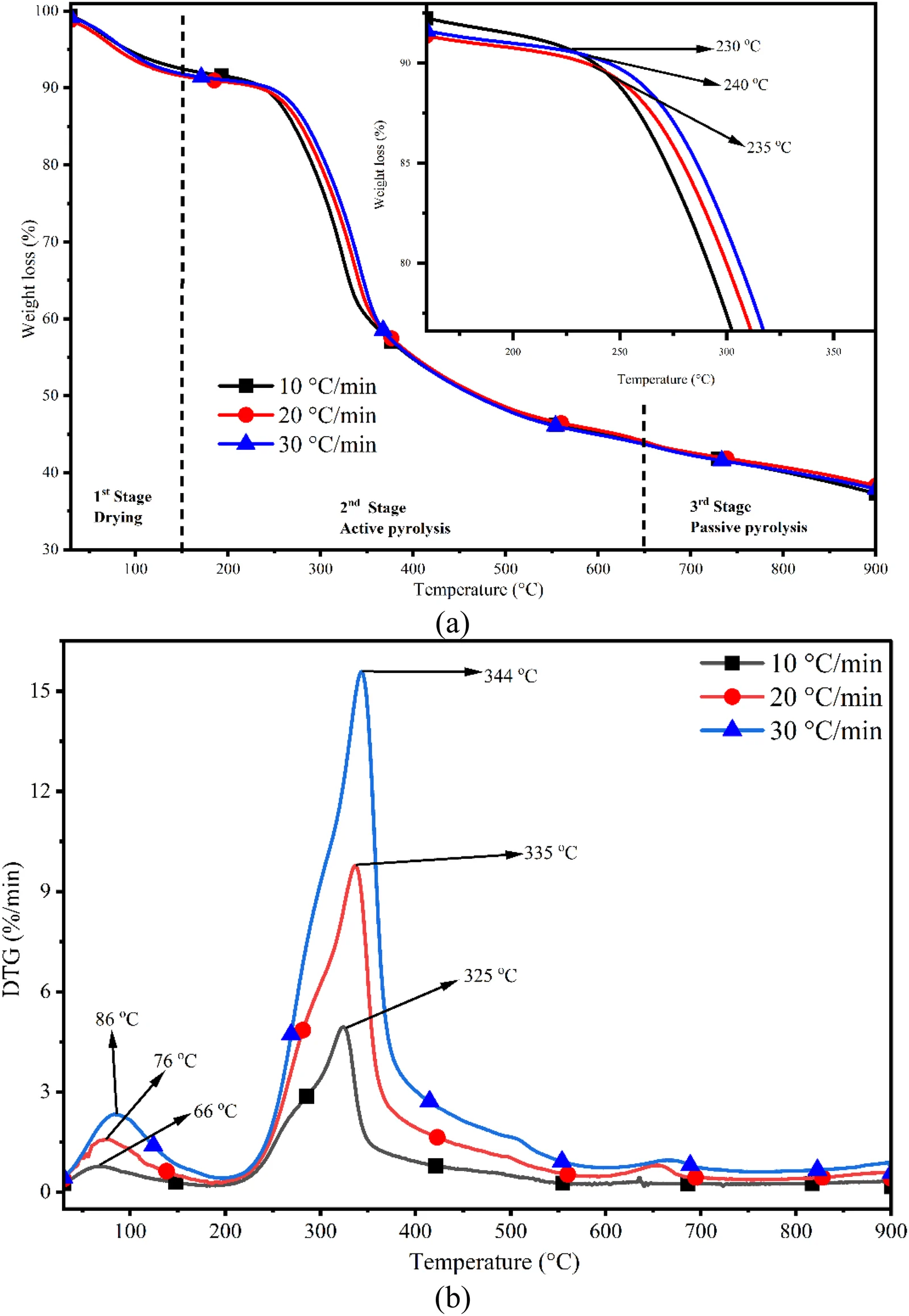
Thermal decomposition of TXT (a) TGA and (b) DTG at 10, 20 and 30 °C min^−1^ heating rates.

The thermogravimetric (TGA) profiles of TXT obtained at heating rates of 10, 20, and 30 °C min^−1^ are presented in Fig. 4(a and b). The TGA curves exhibit three distinct degradation stages: dehydration (30–150 °C), active devolatilization (150–650 °C), and char formation (>650 °C), indicating the complex thermal decomposition behaviour of TXT. Singh *et al.* (2026) studied the Kinetic analysis and pyrolysis behaviour of Effluent Treatment Plant Sludge (ETPS) and reported that the TGA thermograph exhibits three distinct degradation stages: dehydration, active devolatilization, and char formation.^29^ Although the degradation pathways remain similar at all heating rates, increasing the heating rate shifted the decomposition profiles toward higher temperatures due to thermal lag and reduced heat transfer within the sample.^33^ The results confirmed (Fig. 4a) that the first stage (30–150 °C) is associated with the removal of physically adsorbed moisture and low-boiling volatile compounds. During this stage, the sample experienced a relatively small weight loss of approximately 10–12%, and the TGA curves across all heating rates were nearly overlapping, indicating that moisture evaporation was only marginally influenced by the heating rate. The slightly higher residual mass observed at 30 °C min^−1^ suggests incomplete moisture removal due to the shorter residence time for heat penetration. The second stage (150–650 °C) corresponds to the principal decomposition region, where the most rapid mass loss occurs. This stage corresponds to the thermal degradation of organic constituents, proteins, synthetic fibres, dyes, and other volatile organic compounds present in TXT. Increasing the heating rate shifted the onset and peak decomposition temperatures to higher values, a characteristic behaviour attributed to thermal lag, in which the sample core requires additional energy to attain the same decomposition temperature. The TGA curve obtained at 30 °C min^−1^ was slightly shifted to the right relative to those recorded at 10 and 20 °C min^−1^, indicating a delay in decomposition under rapid heating conditions.

The third stage (>650 °C) corresponds to the gradual decomposition of thermally stable organic structures and the formation of carbonaceous char. In this region, the rate of mass loss decreased considerably because the remaining material consisted mainly of aromatic carbon structures and inorganic mineral constituents. The gradual decline in mass is attributed to the slow degradation of residue compounds, secondary carbonisation reactions, and the decomposition of mineral carbonates. At temperatures above 700 °C, only minor changes in TXT weight were observed, demonstrating the formation of a thermally stable char residue enriched with inorganic ash. The final residual mass varied slightly with heating rate, with approximately 32%, 35%, and 40% remaining at heating rates of 10, 20, and 30 °C min^−1^. The higher residual mass at higher heating rates indicates greater solid residue formation under the TGA conditions. The TGA results demonstrate that the heating rate significantly influences the thermal decomposition behaviour of textile sludge by delaying the degradation process and increasing the residual char yield. These observations are consistent with the thermal lag phenomenon commonly reported during biomass and sludge pyrolysis and provide valuable information for selecting appropriate operating conditions for char production.

The DTG curves of TXT at heating rates of 10, 20, and 30 °C min^−1^ (Fig. 4b) exhibit two major decomposition peaks corresponding to moisture evaporation and active devolatilization. The first DTG peaks occurred at 66, 76, and 80 °C, corresponding to the removal of free and bound moisture. The shift of this peak towards higher temperatures with increasing heating rate is attributed to thermal lag and reduced heat transfer efficiency. The second and dominant DTG peak appeared at 325, 335, and 344 °C for heating rates of 10, 20, and 30 °C min^−1^, indicating the maximum decomposition rate of the organic fraction, proteins, dyes, and other volatile compounds. The progressive shift in the peak temperature confirms that higher heating rates delay thermal degradation, as there is insufficient time for uniform heat penetration throughout the sample. It was noticed that beyond 550 °C, the DTG curves gradually approached zero, indicating slow decomposition of thermally stable aromatic carbon structures and secondary char-forming reactions. DTG results demonstrate that increasing the heating rate delays decomposition while maintaining a similar degradation mechanism.

### 3.4. FTIR analysis of TXT

**Fig. 5:**
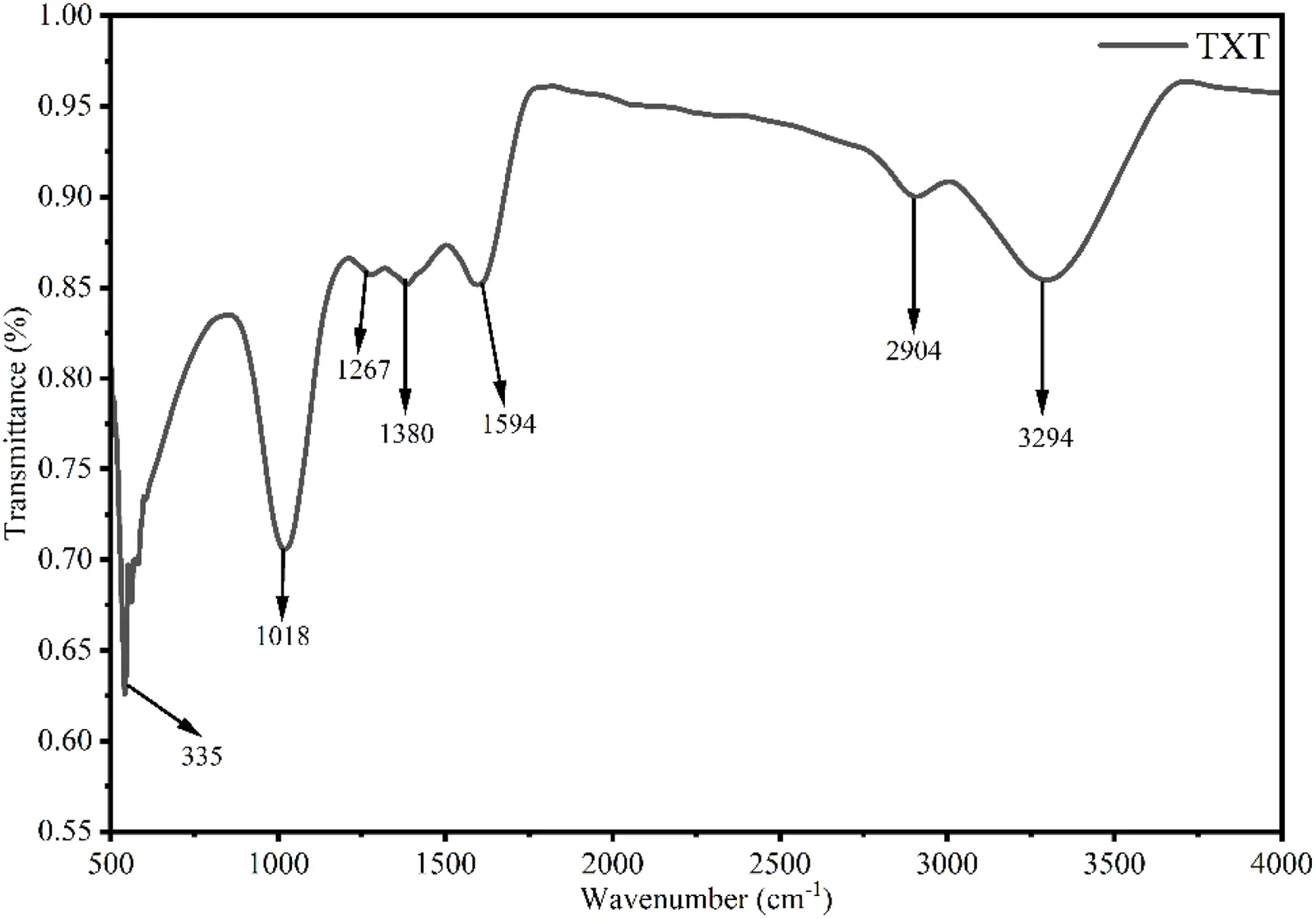
FTIR spectrum of TXT showing the characteristic functional groups at different wavenumbers.

FTIR spectra of TXT were plotted as a function of wavenumber and transmittance, as shown in Fig. 5. The FTIR spectrum of TXT revealed diverse functional groups, consistent with its heterogeneous organic and inorganic composition. The broad absorption band at 3294 cm^−1^ corresponds to O–H stretching vibrations, indicating the presence of hydroxyl groups associated with moisture, alcohols, or phenolic compounds.^18^ The weak peak at 2904 cm^−1^ is attributed to aliphatic C–H stretching of methyl and methylene groups, suggesting the presence of organic constituents.^18^ The absorption band at 1594 cm^−1^ is assigned to aromatic C=C stretching and/or amide (C=O) vibrations, indicating aromatic structures and proteinaceous materials.^29^ Peaks at 1380 cm^−1^ and 1267 cm^−1^ are associated with C–H bending and C–O stretching of phenolic, ester, and ether functional groups.^29^ The prominent peak at 1018 cm^−1^ corresponds to Si–O–Si/C–O stretching, indicating the presence of silicates, polysaccharides, and alcohol groups.^34^ The low-wavenumber band at 335 cm^−1^ may be associated with metal–oxygen vibrations, confirming the presence of inorganic mineral constituents in the textile waste sludge.^18^

### 3.5. Metal content analysis of TXT ash

The mineral composition of TXT ash exhibits a distinct distribution of inorganic oxides that plays a crucial role in determining its thermochemical behaviour, energy recovery potential, and operational performance during thermochemical conversion processes. The presence and relative abundance of these mineral constituents can influence ash melting characteristics, slagging and fouling tendencies, catalytic activity, and conversion efficiency during pyrolysis.^29^ The composition of the major mineral and metal oxides identified in the TXT ash is presented in Table 5. The mineral composition of TXT showed a high abundance of alkali and alkaline-earth metals, which can significantly influence its thermal decomposition and ash behaviour. K_2_O (30.11 wt%) was the dominant oxide, followed by SiO_2_ (21.34 wt%), P_2_O_5_ (16.23 wt%), CaO (11.45 wt%), and Na_2_O (10.04 wt%), while lower concentrations of MgO (7.52 wt%), Fe_2_O_3_ (1.25 wt%), Al_2_O_3_ (0.89 wt%), SO_3_ (0.85 wt%), and Mn_3_O_4_ (0.32 wt%) were detected. High K_2_O and Na_2_O contents are known to promote char gasification and influence ash melting behaviour, whereas a substantial SiO_2_ concentration enhances ash stability by forming silicate phases.^35^ The presence of CaO and MgO can catalyse devolatilisation and secondary cracking reactions during pyrolysis, while phosphorus-containing minerals contribute to char formation and nutrient recovery potential. These oxides are well known for their catalytic role in thermochemical conversion, as they facilitate devolatilisation, lower the activation energy of decomposition reactions, and enhance secondary reactions such as tar cracking and deoxygenation.^36^ It can improve syngas production, increase char reactivity, and enhance the overall efficiency of the pyrolysis process.^37^ The high volatility of alkali and alkaline earth metal oxides, together with their propensity to form low-melting eutectic compounds with silicates and phosphates, can adversely affect reactor operation. These interactions promote slagging, fouling, ash deposition, and bed agglomeration, particularly during combustion and fluidised-bed thermochemical processes.^29^ The relatively high P_2_O_5_ content (16.23 wt%) indicates that TXT is rich in phosphorus-containing minerals. During thermochemical conversion, phosphorus can react with alkali metals, particularly potassium, to form potassium phosphates, which may influence ash melting behaviour and contribute to slagging under certain conditions. Conversely, phosphorus can also interact with calcium to form thermally stable calcium phosphate phases, thereby reducing alkali mobility and partially mitigating ash-related operational issues.^37^ The MgO content (7.52 wt%) contributes to the ash's basic nature and can promote the formation of high-melting magnesium silicates. Its beneficial effect is likely offset by the substantially higher concentrations of K_2_O (30.11 wt%) and Na_2_O (10.04 wt%), which dominate ash chemistry and increase the tendency for slagging and fouling during thermochemical conversion.^38^ The relatively high SiO_2_ content (21.34 wt%) suggests improved ash thermal stability. Its interaction with the abundant K_2_O (30.11 wt%) and Na_2_O (10.04 wt%) can promote the formation of low-melting alkali silicates. This reduces the ash fusion temperature and increases the risk of slagging and fouling. The mineral composition of TXT indicates that high alkali and alkaline-earth metal contents can catalyse devolatilisation and char conversion during pyrolysis, while increasing the likelihood of ash deposition, bed agglomeration, and corrosion in high-temperature thermochemical processes.^38^

**Table 5:** Mineral and metal composition analysis along with slagging and fouling indices

Mineral	TXT (%, db)	ETPS (%, db)^29^
Al_2_O_3_	0.89	0.64
CaO	11.45	12.04
Fe_2_O_3_	1.25	0.39
K_2_O	30.11	28.56
MgO	7.52	9.33
Mn_3_O_4_	0.32	0.59
Na_2_O	10.04	11.72
P_2_O_5_	16.23	18.41
SO_3_	0.85	1.87
SiO_2_	21.34	16.45
Total	100	100
Base-to-acid ratio	1.56	1.75
Alkali index (kg GJ^−1^)	33.46	10.10
Iron calcium ratio	0.11	0.032
Total alkali	40.15	40.28

The base-to-acid ratio of 1.56 indicates that the ash of TXT is moderately basic, suggesting a potential tendency for slagging due to the presence of alkaline oxides. The alkali index of 33.46 kg GJ^−1^, which is substantially higher than the commonly accepted threshold of 0.34 kg GJ^−1^, indicates a very high risk of ash deposition, fouling, and slagging during thermochemical conversion.^39^ The iron-to-calcium ratio of 0.11 suggests that iron is present in relatively low amounts compared with calcium, limiting its ability to stabilise alkali species by forming high-melting-point compounds.^39^ The mineral composition of TXT is expected to promote ash-related operational challenges, particularly under high-temperature combustion or gasification conditions. The total alkali content of TXT was 40.15 wt%, indicating a high concentration of alkali metals in the ash. Such a high alkali content can enhance catalytic activity during pyrolysis by promoting devolatilization and char conversion.^29^ It increases the likelihood of ash-related problems, including slagging, fouling, and bed agglomeration during high-temperature thermochemical processes. The mineral profile suggests that TXT has significant catalytic potential in thermochemical conversion, though its elevated alkali content may increase the risk of ash deposition and slagging in high-temperature applications.

### 3.6. Kinetic analysis

The kinetic behaviour of TXT during thermal decomposition was investigated using five model-free approaches: Kissinger–Akahira–Sunose (KAS), Distributed Activation Energy Model (DAEM), Ozawa–Flynn–Wall (OFW), Tang (TG), and Vyazovkin (VZ). The kinetic analysis of TXT was performed at multiple heating rates (10, 20, and 30 °C min^−1^) because these models only work with dynamic heating rates. The kinetic analysis (apparent activation energy, *R*^2^, and frequency factor) of TXT is listed in Table 6. The kinetic parameters were evaluated over the conversion range of *α* = 0.1–0.8, based on the best fit.^40^ At the higher conversion level (*α* = 0.8), the experimental data showed relatively poor fitting with the kinetic models, as reflected by the lower correlation coefficient, which may be attributed to the increased complexity of the decomposition reactions at the final stage of thermal conversion. Also, conversion values below *α* = 0.1 are often excluded because this region corresponds to the very beginning of mass loss and may not reliably represent the main thermal decomposition process. At very low conversion, small changes in measured mass can produce relatively large changes in the calculated kinetic parameters.^40^ The use of multiple kinetic models provides a broader assessment of the decomposition behaviour, as each method estimates kinetic parameters using a different mathematical approximation. A considerable variation in activation energy with conversion was observed for all the investigated methods, suggesting that thermal decomposition of TXT is kinetically complex and cannot be adequately represented by a single activation-energy value.^40^

**Table 6:** Kinetic analysis of TXT using model-free methods over conversion range 0.1–0.8

Conversion	KAS			DAEM			OFW			TG			VZ	
	*E*_a_ (kJ mol^−1^)	*R* ^2^	*A* (min^−1^)	*E*_a_ (kJ mol^−1^)	*R* ^2^	*A* (min^−1^)	*E*_a_ (kJ mol^−1^)	*R* ^2^	*A* (min^−1^)	*E*_a_ (kJ mol^−1^)	*R* ^2^	*A* (min^−1^)	*E*_a_ (kJ mol^−1^)	Error
0.1	128.65	0.9993	1.40 × 10^11^	154.45	0.9997	2.83 × 10^9^	141.01	0.995	3.10 × 10^16^	109.23	0.9968	1.32 × 10^9^	131.88	2.07 × 10^5^
0.2	188.89	0.9952	2.03 × 10^14^	170.38	0.9989	2.60 × 10^10^	198.08	0.9957	8.60 × 10^18^	132.83	0.9898	1.57 × 10^9^	143.75	2.14 × 10^5^
0.3	193.59	0.9996	1.45 × 10^14^	177.18	0.9953	4.02 × 10^10^	203.18	0.9996	6.36 × 10^18^	135.20	0.9778	1.15 × 10^9^	161.67	1.22 × 10^4^
0.4	203.27	0.9999	4.41 × 10^14^	189.70	0.9998	2.38 × 10^11^	213.16	0.9999	1.86 × 10^19^	141.47	0.9744	2.51 × 10^9^	169.15	1.51 × 10^7^
0.5	228.97	0.9989	3.56 × 10^16^	201.17	0.998	1.16 × 10^12^	239.11	0.999	1.25 × 10^21^	159.22	0.9805	5.85 × 10^10^	166.74	2.25 × 10^5^
0.6	244.19	0.9867	1.24 × 10^17^	238.41	0.9951	6.62 × 10^11^	248.54	0.9861	1.32 × 10^21^	189.69	0.9944	6.89 × 10^13^	183	1.25 × 10^4^
0.7	249.71	0.9949	4.09 × 10^15^	258.12	0.9927	5.43 × 10^11^	246.97	0.9941	1.41 × 10^19^	190.56	0.9900	2.94 × 10^11^	200	0.0016
0.8	267.22	0.9777	1.43 × 10^14^	281.08	0.9999	4.05 × 10^10^	258.80	0.975	2.89 × 10^17^	203.55	0.9889	2.44 × 10^10^	205	0.0011
Average	213.03		2.06 × 10^16^	208.81		3.39 × 10^11^	218.61		3.28 × 10^20^	157.72		8.66 × 10^12^	170.14	

KAS model predicted activation energies ranging from 128.65 kJ mol^−1^ at conversion (*α* = 0.1) to 267.22 kJ mol^−1^ at *α* = 0.8, with an average *E*_a_ of 213.03 kJ mol^−1^. From Table 6 and it was observed that as *E*_a_ increased, conversion increased. For example, *E*_a_ increased from 128.65 kJ mol^−1^ at *α* = 0.1 to 188.89 and 193.59 kJ mol^−1^ at *α* = 0.2 and 0.3, before reaching 228.97–267.22 kJ mol^−1^ in the conversion range of *α* = 0.5–0.8. This behaviour indicates that the energy requirement for decomposition increases as the reaction progresses.^9^ The frequency factor (*A*) represents the frequency of effective molecular collisions leading to thermal decomposition. Its increase with conversion indicates changes in reaction kinetics as decomposition progresses. The wide variation in A suggests that different reaction pathways and degradation steps become dominant at different conversion levels, confirming the complex nature of TXT decomposition.^41^ The KAS model exhibited *R*^2^ values ranging from 0.9777 to 0.9999, *R*^2^ demonstrating a strong linear relationship for most conversion levels.^41^ DAEM results showed a broadly similar conversion-dependent trend. The activation energy increased from 154.45 kJ mol^−1^ at *α* = 0.1 to 281.08 kJ mol^−1^ at *α* = 0.8, producing an average *E*_a_ of 208.81 kJ mol^−1^. At intermediate conversions of *α* = 0.3, 0.4, and 0.5, the estimated *E*_a_ values were 177.18, 189.70, and 201.17 kJ mol^−1^. A sharper increase occurred at higher conversions, reaching 238.41, 258.12, and 281.08 kJ mol^−1^ at *α* = 0.6, 0.7, and 0.8. The increase in activation energy at higher conversions (0.6–0.8) indicates that the readily degradable fractions were consumed during the earlier stages, leaving more thermally resistant components. Greater energy was required for further decomposition, suggesting increased reaction complexity and the involvement of multiple degradation pathways at advanced conversion stages.^41^ This systematic increase supports the presence of conversion-dependent reaction energetics. The *R*^2^ values remained high, ranging from approximately 0.9927 to 0.9999, indicating an excellent fit to the experimental data. The frequency factor changed markedly with conversion, from 2.83 × 10^9^ min^−1^ at *α* = 0.1 to a reported value of 4.05 × 10^10^ min^−1^ at *α* = 0.8. The extremely large pre-exponential factors obtained at high conversion should not be interpreted as direct molecular collision frequencies; they reflect the strong kinetic compensation effect associated with conversion-dependent apparent activation energies.

The OFW method yielded the highest average activation energy among the investigated models, at 218.61 kJ mol^−1^. The individual values increased from 141.01 kJ mol^−1^ at conversion *α* = 0.1 to 258.80 kJ mol^−1^ at 0.8. *E*_a_ increased to 198.08, 203.18, and 213.16 kJ mol^−1^ at conversion 0.2, 0.3, and 0.4, followed by 239.11 and 248.54 kJ mol^−1^ at *α* = 0.5 and 0.6. OFW results closely resemble the trend observed using KAS and DAEM. The corresponding frequency factor ranged from 3.10 × 10^16^ to 1.32 × 10^21^ min^−1^, with an average of 3.28 × 10^20^ min^−1^. *R*^2^ values were generally high, reaching 0.9999 at conversion 0.4 and remaining above approximately 0.975 throughout the investigated conversion range. The close agreement among KAS, DAEM, and OFW is particularly important because these models are mathematically different but independently indicate increasing activation-energy requirements with conversion.^42^ In comparison with these three methods, the TG model predicted lower activation-energy values. *E*_a_ increased from 109.23 kJ mol^−1^ at *α* = 0.1 to 203.55 kJ mol^−1^ at *α* = 0.8, with an average value of 157.72 kJ mol^−1^. It was found that between *α* = 0.1 and 0.4, *E*_a_ increased from 109.23 to 141.47 kJ mol^−1^, followed by a more pronounced increase to 159.22, 189.69, 190.56, and 203.55 kJ mol^−1^ at *α* = 0.5–0.8. The progressive increase in *E*_a_ with conversion indicates that readily degradable fractions decomposed during the initial stages, whereas more thermally resistant fractions became dominant at higher conversions. Greater energy was required to continue decomposition, reflecting changes in the controlling reactions and suggesting a complex, multistep thermal degradation mechanism.^43^ The corresponding frequency factor ranged from 1.15 × 10^9^ to 6.89 × 10^13^ min^−1^, with an average of 8.66 × 10^12^ min^−1^. The *R*^2^ value was greater than 0.95, indicating an acceptable/good linear fit. Although the absolute *E*_a_ values obtained from TG were lower than those calculated using KAS, DAEM, and OFW, the increasing trend in *E*_a_ with conversion remained evident.

The VZ method yielded activation energies ranging from 131.88 to 205 kJ mol^−1^, with an average value of 170.14 kJ mol^−1^. *E*_a_ increased from 131.88 kJ mol^−1^ at *α* = 0.1 to 143.75, 161.67, and 169.15 kJ mol^−1^ at *α* = 0.2–0.4. At higher conversion, the values increased to 183, 200, and 205 kJ mol^−1^ at *α* = 0.6, 0.7, and 0.8. The numerical error reported for the VZ optimisation remained relatively small at most low-to-intermediate conversion levels but increased to 0.0016 and 0.3011 at *α* = 0.7 and 0.8, respectively. The progressive increase in *E*_a_ with conversion indicates a shift toward reactions with higher apparent energy barriers as thermal decomposition proceeds. The increased VZ optimisation error at *α* = 0.7–0.8 suggests reduced numerical reliability at advanced conversion, potentially arising from greater kinetic complexity and increased sensitivity of the optimisation procedure in the final decomposition region.^44^ Comparison of the average activation energies gives the order OFW (218.61 kJ mol^−1^) > KAS (213.03 kJ mol^−1^) > DAEM (208.81 kJ mol^−1^) > VZ (170.14 kJ mol^−1^) > TG (157.72 kJ mol^−1^). Importantly, KAS, DAEM, and OFW produced relatively comparable average *E*_a_ values, whereas TG and VZ generated lower values due to mathematical differences.^45^ Nevertheless, all five approaches showed a general increase in *E*_a_ as conversion increased. This consistent dependence on conversion is more informative than any single average *E*_a_ value and indicates that different reaction contributions dominate at different stages of TXT conversion.

At the initial conversion stage, the relatively low *E*_a_ values indicate that decomposition can proceed with a lower energy requirement. As conversion increases, the increasing *E*_a_ indicates that the remaining material requires more thermal energy for further conversion. In a multicomponent system, such behaviour can be associated with sequential and overlapping degradation of fractions with different thermal stabilities.^45^ The progressive increase in apparent *E*_a_ with conversion has an important chemical significance. At low conversion, the decomposition is dominated by relatively labile and thermally accessible organic fractions, which can undergo dehydration, volatilisation, and cleavage of weaker chemical bonds at comparatively lower energy requirements. As conversion proceeds, these readily degradable fractions are progressively depleted, and the remaining solid becomes enriched in more thermally resistant structures, including aromatic and condensed carbonaceous constituents, as well as mineral-associated organic matter. Consequently, a higher energy input is required to achieve further conversion. Thus, the increasing *E*_a_ should not be interpreted simply as a gradual increase in temperature requirement; rather, it reflects a change in the relative contribution of different overlapping reactions and feedstock fractions during pyrolysis.

The considerable variation in the frequency factor provides evidence of changing apparent kinetic behaviour throughout conversion (Table 6). Since the frequency factor and apparent activation energy are Arrhenius parameters, changes in the estimated activation energy are accompanied by substantial changes in the corresponding frequency factor.^46^ The extremely broad range of A for DAEM should be considered alongside the activation energy and the mathematical formulation, rather than interpreted independently as a direct measure of reaction frequency. The kinetic analysis demonstrates that TXT undergoes a complex, conversion-dependent thermal decomposition process.^46^ Ali *et al.* (2025) demonstrated that textile sludge combustion is highly conversion-dependent. The activation energy (*E*_a_) decreased markedly from 1027.86 to 147.31 kJ mol^−1^ using Friedman, while KAS and OFW decreased from 1134.95 to 151.61 and 1087.50 to 155.49 kJ mol^−1^, respectively, over the conversion range (0.20–0.80). The combined kinetic model estimated *E*_a_ of 180.2 ± 5.5 kJ mol^−1^ (*R*^2^ = 0.905). M-DAEM identified four pseudo-components, with *E*_a_ values ranging from 144.5 to 370.50 kJ mol^−1^.^47^ Singh *et al.* (2026) confirmed the complex, multistage pyrolysis behaviour of Effluent Treatment Plant Sludge (ETPS). Across the conversion range from (0.1–0.8), activation energies varied from 139 to 350 kJ mol^−1^, with average values of 185–225 kJ mol^−1^. Lower conversions required 140–200 kJ mol^−1^, while intermediate conversions increased to approximately 250–300 kJ mol^−1^, indicating stronger bond degradation and mineral–organic interactions. All kinetic models showed high correlation (*R*^2^ > 0.98), confirming reliable kinetic predictions and heterogeneous decomposition behaviour.^29^

The comparative analysis presented in Table 7 indicates that the apparent activation energy (*E*_a_) of TXT obtained in the present study is higher than that reported for leather and textile industry sludge (182.77 kJ mol^−1^ by KAS), mixed waste fabric pieces (185.10 kJ mol^−1^ by KAS), and mustard straw (201.80 kJ mol^−1^ by KAS), but lower than the DAEM-derived Ea reported for effluent treatment plant (ETP) sludge (220.49 kJ mol^−1^). KAS, OFW, and DAEM methods yielded comparable values of 213.03, 208.81, and 218.61 kJ mol^−1^, confirming the reliability of the iso-conversional kinetic models for evaluating the pyrolysis of textile waste. In contrast, TG (157.72 kJ mol^−1^) and VZ (170.14 kJ mol^−1^) methods predicted comparatively lower activation energies, reflecting differences in mathematical formulations and optimisation procedures.^29^ The higher *E*_a_ of textile waste compared with mixed textile residues and industrial sludge suggests the presence of thermally stable polymeric constituents and stronger chemical bonds that require greater energy for decomposition. The close agreement among KAS, OFW, and DAEM values (within 5%) demonstrates the robustness of the kinetic evaluation, while the deviations in TG and VZ highlight the sensitivity of the activation energy to the selected kinetic model. The differences in apparent activation energy obtained from the kinetic models arise from the mathematical treatment of the temperature–conversion relationship, the approximations used to evaluate the temperature integral, and the treatment of experimental conversion data. In the present study, KAS, OFW, and DAEM produced relatively comparable average activation energies of 213.03, 208.81, and 218.61 kJ mol^−1^, respectively, whereas the Tang and VZ approaches yielded lower values of 157.72 and 170.14 kJ mol^−1^, respectively. Such differences do not necessarily indicate contradictory reaction behaviour; rather, they reflect the sensitivity of apparent kinetic parameters to the formulation and numerical treatment adopted by each method. Importantly, the five methods showed the same overall increase in *E*_a_ with conversion, suggesting that the conversion-dependent trend is more physically meaningful than comparison of a single average *E*_a_ value.

**Table 7:** Comparison of average apparent activation energy against different feedstock using different model-free methods

Feedstock name	Heating rate (°C min^−1^)	KAS (kJ mol^−1^)	OFW (kJ mol^−1^)	DAEM (kJ mol^−1^)	TG (kJ mol^−1^)	VZ (kJ mol^−1^)	References
TXT	10, 20 and 30	213.03	208.81	218.61	157.72	170.14	Present study
Mixed waste fabric pieces	5, 10, 20 and 40	185.10	185.80	—	—	—	50
Leather and tex-tile Industry sludge	5, 10, 15 and 20	182.77	170.63	—	—	—	13
Mustard straw	10, 15, 20, 30, and 40	201.80	202.19	—	—	202.12	51
Effluent treatment plant sludge	10, 20, and 30	139.45	189.65	220.49	—	222.49	29

**Fig. 6:**
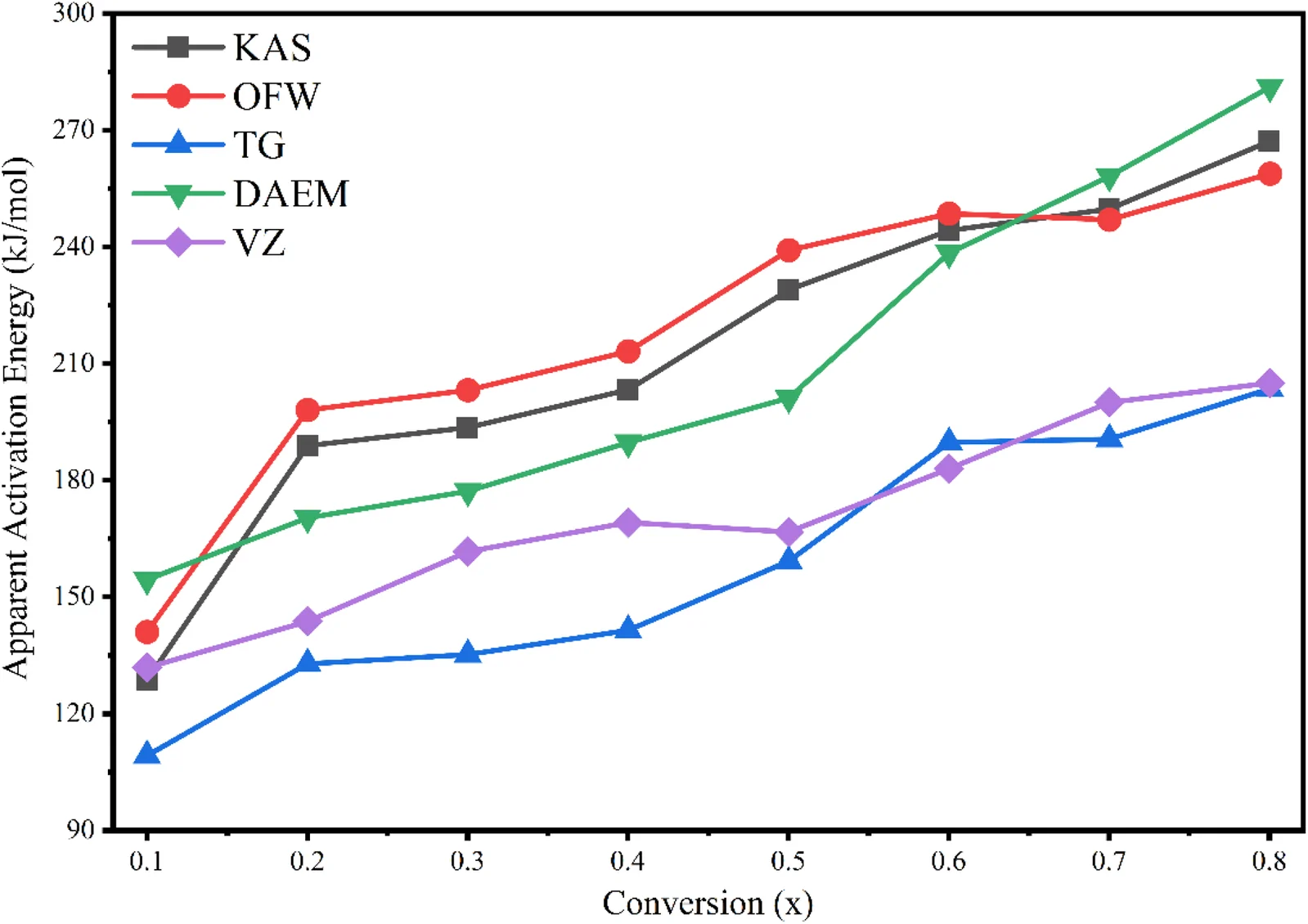
Variation of activation energy calculated using model-free methods against conversion value.

Fig. 6 shows the variation in apparent activation energy with conversion. The apparent activation energy (*E*_a_) showed a clear conversion-dependent variation across all five kinetic models, indicating the complex, multistep nature of the thermal decomposition process.^46^ At a lower conversion (*α* = 0.1), relatively low *E*_a_ values suggest that decomposition is initially dominated by readily degradable fractions that require less energy.^46^ Further, with increasing conversion, *E*_a_ progressively increased, indicating a shift toward the degradation of comparatively thermally resistant fractions. KAS, OFW, and DAEM generally predicted higher *E*_a_ values than TG and VZ, although all models displayed a similar increasing trend (Table 6). The close agreement between KAS and OFW over most conversion levels demonstrates consistency between these two conversional approaches. Also, at *α* > 0.5, an obvious increase in DAEM-derived *E*_a_ was observed, reaching approximately 281 kJ mol^−1^ at *α* = 0.8. The non-constant *E*_a_ profile confirms that the decomposition cannot be represented by a single kinetic barrier and likely involves sequential and overlapping reactions with different apparent energy requirements.^48^ The activation energy (*E*_a_) varies across kinetic models because each method employs a different mathematical formulation, approximations, and procedures for evaluating the temperature–conversion relationship. KAS, OFW, and DAEM generally predict higher *E*_a_ values, whereas TG and VZ provide comparatively lower estimates.^11,49^ Despite these quantitative differences, all models show an overall increase in *E*_a_ with conversion, indicating consistent kinetic behaviour. The differences in absolute *E*_a_ values primarily arise from model-specific computational assumptions, while the increasing trend reflects the underlying conversion-dependent decomposition.

The observed mechanism transition is broadly consistent with previous studies of heterogeneous sludge and textile-derived materials, in which the apparent reaction behaviour changes with conversion as different organic fractions and transport limitations become dominant at different stages. Wang *et al.* (2024) reported a three-dimensional diffusion-controlled model for co-pyrolysis of waste textiles with Ca-rich and Fe-rich sludge, demonstrating the importance of mass-transfer effects in mineral-containing textile-derived feedstocks.^4^ Wahab *et al.* (2022) evaluated sludge pyrolysis using the KAS, FWO, and Friedman methods over conversion degrees of 0.1–0.8. The average activation energies for non-catalytic pyrolysis were 182.77, 170.63, and 175.83 kJ mol^−1^, respectively. The corresponding kinetic plots showed strong linearity, with *R*^2^ values generally above 0.93. Addition of Al_2_O_3_ and γ-zeolite reduced the activation energy, with average KAS values of 135.92 and 123.10 kJ mol^−1^, respectively, demonstrating their catalytic effectiveness.^13^

### 3.7. Master plot analysis

The average activation energy (*E*_a_) obtained using the DAEM and VZ method for textile waste sludge was employed to evaluate the thermal decomposition mechanism according to the master-plot approach proposed by Criado *et al.* (1989).^12^ The theoretical reference curves were generated using eqn (19) based on the corresponding (*f*(*x*)) and (*g*(*x*)) functions listed in Table 3. These theoretical master curves were subsequently compared with the experimental data. The reaction model that best fit the experimental curve was considered the most probable solid–state reaction mechanism governing thermal decomposition. As presented in Table 3, the algebraic reaction models are classified into five major categories: *F*_*n*_, *D*_*n*_, *R*_*n*_, *P*_*n*_, and *A*_*n*_. The theoretical master curves include diffusion-controlled mechanisms (*D*_1_–*D*_4_), reaction-order mechanisms (*F*_1_–*F*_3_), nucleation-and-growth mechanisms (*A*_2_–*A*_4_), phase-boundary-controlled mechanisms (*R*_1_–*R*_3_), and power-law mechanisms (*P*_2_–*P*_4_). The experimental (*Z*(*x*)) values were determined at a heating rate of 10 °C min^−1^. Table 8 shows the conversion-dependent reaction mechanism families identified from Criado master-plot analysis of TXT pyrolysis.

**Table 8:** Conversion-dependent reaction mechanism families identified from Criado master-plot analysis of TXT pyrolysis

Model family	Codes	Interpretation over conversion range (0.1–0.8)
Diffusion	*D*_1_–*D*_4_	Most relevant at low conversion (0.1–0.3); contribution decreases as conversion progresses
Reaction-order	*F*_1_–*F*_3_	Becomes increasingly important from intermediate conversion and is particularly relevant above conversion =0.5
Nucleation & growth	*A*_2_–*A*_4_	Limited at early conversion; becomes increasingly relevant at intermediate-to-high conversion, particularly conversion >0.5
Phase boundary	*R*_1_–*R*_3_	Do not provide the dominant description over the complete conversion range; it may contribute locally, but does not explain the overall experimental trajectory
Power law	*P*_2_–*P*_4_	Generally, shows weaker correspondence with the experimental trajectory than the diffusion, reaction-order and nucleation families

**Fig. 7:**
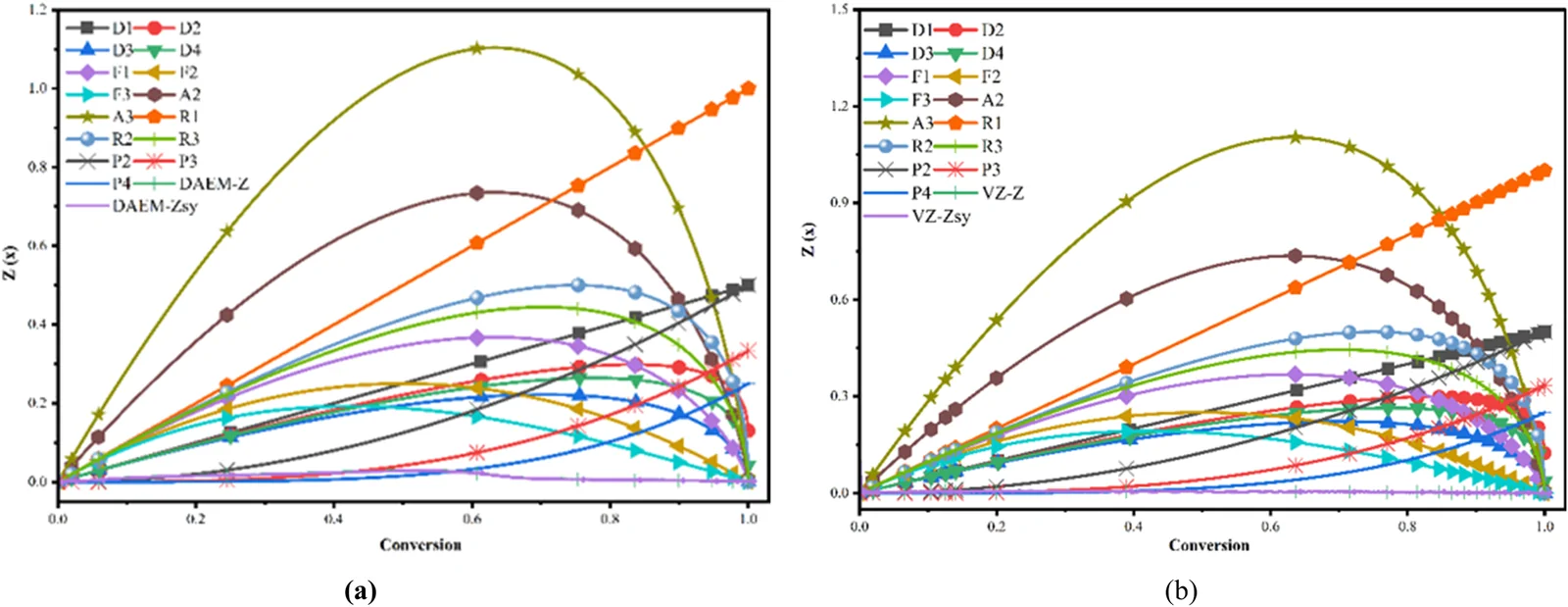
Theoretical and experimental master plots based on the Criado method (a) DAEM and (b) VZ.

Fig. 7(a and b) supports a most-probable mechanism that the conversion range (0.1–0.2), DAEM and VZ-based experimental curves are located predominantly within or very close to the region occupied by the diffusion-controlled models (*D*_1_–*D*_4_) (Table 8). This indicates that transport phenomena contribute substantially to the initial stage of thermal decomposition.^52^ At this stage, the TXT matrix remains relatively intact and contains readily decomposable, volatile organic constituents. Thermal degradation generates volatile products that must migrate through the partially undecomposed solid matrix.^52^ Consequently, heat and mass transfer limitations can contribute significantly to the apparent conversion behaviour. This interpretation is consistent with the observed experimental master-plot behaviour.^53^ The manuscript identifies diffusion-controlled behaviour as dominant at lower conversion. Among the diffusion models, *D*_1_–*D*_4_ represent different mathematical descriptions of diffusion through the reacting solid, rather than four completely different chemical reactions.^53^ Therefore, the experimental proximity to the D-family should be interpreted primarily as evidence for transport-controlled decomposition, rather than as definitive evidence that a particular diffusion geometry is responsible.^52^ From the perspective of char formation, this stage is important because the solid carbon precursor is largely retained. A limited mass has been removed, so the potential char yield remains comparatively high. This behaviour is consistent with the relatively high experimental char yield of 51.20 wt% at 450 °C. The manuscript reports that increasing pyrolysis severity progressively removes volatile organic matter and decreases the final char yield.^54^

Further increasing the conversion range from 0.2 to 0.3, the experimental curves begin to move away from a purely diffusion-controlled description. The diffusion–model curves remain relevant, but increasing proximity to the reaction-order (F1–F3) and the nucleation-related curves become apparent (Table 8). This behaviour indicates that thermal decomposition is increasingly influenced by the intrinsic transformation of the organic constituents rather than solely by the transport of the initially generated volatiles.^55^ The progressive removal of readily volatile fractions exposes less-reactive material and begins to alter the physical structure of the sludge matrix.^10^ The increasing conversion corresponds to progressive decomposition and volatilisation of the more reactive organic constituents.^53^ Consequently, volatile matter is transferred from the solid phase to gaseous/condensable products, while the less-reactive carbonaceous and inorganic fractions become increasingly concentrated in the remaining solid.^55^ Thus, this conversion region represents an important stage in the initial reduction of potential char yield through devolatilisation, while simultaneously initiating carbon enrichment of the remaining solid.^22^ Conversion range (0.2–0.3) can be regarded as a transition from predominantly transport-controlled behaviour toward mixed transport-chemical-reaction control.^56^

In the range of 0.3–0.4, the experimental response shows departure from the diffusion-only curves (Table 8). The F-type reaction-order models become increasingly relevant, while some nucleation-and-growth models also approach the experimental response. This behaviour suggests that the decomposition of the more reactive organic fractions is progressing and that the reaction rate increasingly depends on the amount and nature of the remaining reactive material.^53^ In a heterogeneous textile sludge, this stage can involve overlapping decomposition of different organic constituents rather than a single elementary reaction. The increasing importance of the F-family indicates a transition toward chemically controlled conversion, although the continued proximity of diffusion-related curves demonstrates that transport effects have not completely disappeared.^53^ This stage can be associated with substantial devolatilisation, fragmentation and transformation of the organic constituents. As volatile products leave the solid, the remaining matrix becomes progressively enriched in fixed carbon and inorganic minerals.^41^ The increasing conversion simultaneously causes loss of solid mass and development of the precursor structure of char. The observed decrease in char yield from 51.20 wt% at 450 °C to 45.78 wt% at 600 °C is consistent with this progressive devolatilization.^22^

At conversion range (0.4–0.5), the experimental curves show a clear transition region between the diffusion-controlled and chemically controlled mechanisms. The agreement with the D-family weakens progressively, whereas the reaction-order and nucleation-and-growth families become more relevant. This transition can be associated with progressive depletion of the easily decomposable organic fraction and increasing formation of thermally resistant carbonaceous structures.^53^ As conversion proceeds, the remaining solid becomes increasingly enriched in carbonaceous and inorganic components. Conversion level (0.5) represents an important mechanistic transition point in the pyrolysis process. At this stage, carbonisation becomes progressively more important. Dehydration, dehydrogenation and removal of oxygen-containing functionalities contribute to the transformation of the remaining organic matrix into a more condensed carbonaceous structure.^57^ Consequently, although the total solid mass continues to decrease, the chemical stability and carbon concentration of the residual char increase.^58^ This interpretation is supported by the elemental results: carbon content increases from 41.04 wt% at 450 °C to 42.92 wt% at 600 °C, while hydrogen decreases from 2.12 to 0.85 wt%.

In the high-conversion range (0.5–0.6), the contribution of diffusion-controlled mechanisms diminishes. The experimental response increasingly approaches the F1–F3 reaction-order models and A2–A4 nucleation-and-growth models. This indicates that the later stage of conversion is increasingly controlled by transformations occurring within the residual solid rather than by the outward transport of volatile products alone.^9^ The residual matrix contains increasingly carbonised structures and mineral-associated components, which require greater thermal severity for further conversion.^53^ The kinetic results support this interpretation, as the activation energy varies substantially with conversion, indicating that the process cannot be represented by a single invariant reaction mechanism.^9^ This region is relevant to char stabilisation. The readily decomposable organic fraction has already been removed, leaving a progressively carbonised matrix. As conversion proceeds, additional volatile matter is removed, increasing the relative concentration of carbon and mineral matter in the residual char.^58^ The experimentally observed increase in ash content from 43.0 wt% at 450 °C to 47.95 wt% at 600 °C and 48.14 wt% at 700 °C supports the progressive concentration of the inorganic fraction as organic matter is removed.

The conversion range (0.6–0.7) indicated that the experimental curves exhibited their strongest departure from simple diffusion-controlled models and greater correspondence with reaction-order and nucleation-and-growth behaviour. The increasing contribution of nucleation-related models may reflect the progressive formation and growth of new carbonaceous structures within the residual solid.^46^ However, it is important not to interpret this as proof of a specific molecular nucleation reaction. In a heterogeneous solid such as textile sludge, the A2–A4 models should instead be interpreted as mathematical descriptions of the observed conversion behaviour associated with progressive structural transformation.^53^ This region represents a chemically and structurally controlled stage of pyrolysis, characterised by depletion of readily reactive constituents and increasing resistance of the remaining carbonaceous matrix.^46^ This region corresponds to the development and stabilisation of the char framework rather than simply the release of volatile matter. The decrease in H/C ratio provides strong supporting evidence: H/C decreases substantially with increasing pyrolysis severity, reaching 0.20 at 700 °C. At the reactor scale, this increasing carbonisation is accompanied by a continued decrease in char yield. The measured yield decreases to 44.83 wt% at 700 °C. However, the lower mass yield does not necessarily indicate poorer char quality; rather, it reflects greater removal of volatile constituents and greater transformation/stabilisation of the remaining carbonaceous fraction.

In the conversion range (0.7–0.8), the experimental curves increasingly approach the reaction-order and nucleation-related regions, while agreement with the simple diffusion models becomes comparatively weaker. At this stage, the process involves converting the most thermally resistant fraction of the sludge.^59^ The remaining solid is increasingly carbonised, aromatic and mineral-rich. The additional conversion requires greater structural rearrangement and decomposition of thermally stable carbonaceous components.^58^ This interpretation is consistent with the manuscript's observation that the higher-conversion region corresponds to the formation of a more resistant carbonaceous matrix and increasing contributions from reaction-order and nucleation-related mechanisms.^53^ Further thermal treatment causes additional devolatilisation and secondary reactions, resulting in continued loss of solid mass. Consequently, the final char yield reaches its lowest measured value of 43.53 wt% at 800 °C. Cai *et al.* (2019) investigated textile dyeing sludge using TGA and master-plot analysis. For pure TDS, three mechanisms were identified: D2 diffusion (144.3–227.5 °C; *E*_a_ = 119.05 kJ mol^−1^), followed by F3 third-order reaction (227.5–351.2 °C; *E*_a_ = 182.63 kJ mol^−1^) and F3 (351.2–645.8 °C; *E*_a_ = 167.60 kJ mol^−1^). The results demonstrate that textile-sludge thermal conversion proceeds through multiple, stage-dependent mechanisms rather than a single reaction model.^57^ Wen *et al.* (2021) applied the Master-Plot method to textile dyeing sludge co-combustion and identified F2.4, F1.5, D3 and A1.5 mechanisms across four stages. For 50TDS/50IS, mean activation energies were 176.75, 171.71, 445.84 and 194.10 kJ mol^−1^, respectively. The results demonstrate that textile sludge undergoes conversion-dependent, multistage mechanisms, supporting the interpretation of your Criado plots over the conversion range (0.1–0.8).^60^

Overall, the diffusion-controlled behaviour observed at lower conversion should be considered in relation to the inorganic-rich nature of TXT. The ash contained substantial amounts of K_2_O (30.11 wt%), SiO_2_ (21.34 wt%), P_2_O_5_ (16.23 wt%), CaO (11.45 wt%), and Na_2_O (10.04 wt%), indicating that mineral matter constitutes an important component of the feedstock. During the early stages of pyrolysis, volatile products generated from the decomposition of organic constituents must migrate through the solid matrix before escaping from the particle.^61^ The presence and distribution of mineral matter can modify pore structure, local heat transfer, and mass–transfer pathways and may influence the apparent contribution of diffusion to the overall conversion rate.^61^ In addition, alkali and alkaline-earth minerals can interact with oxygenated organic structures and alter secondary reactions, meaning that the observed diffusion-controlled behaviour represents an apparent transport-reaction response of the heterogeneous sludge rather than diffusion through a purely organic matrix.

### 3.8. Thermodynamic analysis

The thermodynamic parameters of TXT, including enthalpy change (Δ*H*), Gibbs free energy change (Δ*G*), and entropy change (Δ*S*), were determined over the conversion range of *α* = 0.1–0.8 using the activation energies obtained from the KAS, DAEM, OFW, and TG kinetic models. The calculated value of the thermodynamic property is listed in Table 9. These thermodynamic parameters provide information on energy demand and changes in the system's energetic state during different stages of thermal decomposition.^62^ Considerable variation in Δ*H* and Δ*S* with conversion was observed, whereas Δ*G* remained comparatively stable, indicating the complex nature of TXT.^63^ The enthalpy change increased from 123.69 kJ mol^−1^ at *α* = 0.1 to 262.27 kJ mol^−1^ at *α* = 0.8 for the KAS model. The corresponding Δ*G* decreased slightly from 208.09 to 204.46 kJ mol^−1^, whereas Δ*S* increased substantially from −141.57 to 96.96 J mol^−1^. K. The average Δ*H*, Δ*G*, and Δ*S* values obtained using KAS were 208.10 kJ mol^−1^, 205.69 kJ mol^−1^, and 4.04 J mol^−1^. K. The continuous increase in Δ*H* with conversion indicates that the energy required for decomposition increases as pyrolysis progresses.^63^ The negative Δ*S* values at lower conversions suggest a relatively more ordered activated state, while the positive values observed at higher conversions indicate an increase in disorder during the later stages of thermal decomposition. The DAEM model exhibited a substantial conversion-dependent variation in the thermodynamic parameters. Δ*H* increased from 149.50 to 276.13 kJ mol^−1^ between *α* = 0.1 and 0.8. In comparison, Δ*G* decreased only slightly from 207.18 to 204.21 kJ mol^−1^. The entropy change increased from −96.76 J mol^−1^ K at conversion 0.1 to 120.63 J mol^−1^ K at conversion 0.8. The average values of Δ*H*, Δ*G*, and Δ*S* were 203.86 kJ mol^−1^, 205.78 kJ mol^−1^, and −3.23 J mol^−1^. The increasing Δ*H* indicates progressively higher energy requirements at advanced conversion stages, consistent with the involvement of fractions with different thermal resistance.^64^

**Table 9:** Thermodynamic parameters of textile waste sludge were calculated using activation energies obtained from KAS, DAEM, OFW, and Tang method

Conversion	KAS			DAEM			OFW			TG		
	Δ*H* (kJ mol^−1^)	Δ*G* (kJ mol^−1^)	Δ*S* (J mol^−1^ K^−1^)	Δ*H* (kJ mol^−1^)	Δ*G* (kJ mol^−1^)	Δ*S* (J mol^−1^ K^−1^)	Δ*H* (kJ mol^−1^)	Δ*G* (kJ mol^−1^)	Δ*S* (J mol^−1^ K^−1^)	Δ*H* (kJ mol^−1^)	Δ*G* (kJ mol^−1^)	Δ*S* (J mol^−1^ K^−1^)
0.1	123.69	208.09	−141.57	149.50	207.18	−96.76	136.05	207.63	−120.07	104.28	208.90	−175.49
0.2	183.93	206.18	−37.32	165.43	206.69	−69.22	193.13	205.95	−21.50	127.87	207.93	−134.29
0.3	188.63	206.06	−29.23	172.23	206.50	−57.48	198.22	205.82	−12.74	130.24	207.84	−130.17
0.4	198.32	205.82	−12.58	184.75	206.16	−35.91	208.20	205.58	4.39	136.51	207.61	−119.27
0.5	224.01	205.23	31.51	196.21	205.87	−16.19	234.15	205.01	48.87	154.27	207.03	−88.50
0.6	239.24	204.91	57.58	233.45	205.03	47.67	243.59	204.82	65.02	184.73	206.16	−35.94
0.7	244.75	204.80	67.01	253.16	204.63	81.40	242.01	204.85	62.33	185.60	206.14	−34.44
0.8	262.27	204.46	96.96	276.13	204.21	120.63	253.85	204.62	82.56	198.59	205.81	−12.11
Average	208.10	205.69	4.04	203.86	205.78	−3.23	213.65	205.54	13.61	152.76	207.18	−91.27

A similar trend was obtained using the OFW model. The Δ*H* value increased from 136.05 kJ mol^−1^ at conversion 0.1 to 253.85 kJ mol^−1^ at conversion 0.8, whereas Δ*G* varied within a much narrower range, from 207.63 to 204.62 kJ mol^−1^ Δ*S* increased markedly from −120.07 to 82.56 J mol^−1^ K^−1^ over the same conversion interval. The average Δ*H*, Δ*G*, and Δ*S* values were 213.65 kJ mol^−1^, 205.54 kJ mol^−1^, and 13.61 J mol^−1^ K^−1^. Among the investigated models, OFW yielded the highest average Δ*H*, indicating a comparatively greater overall energetic requirement under this kinetic formulation (Table 9). The transition of Δ*S* from negative to positive values indicates substantial changes in the molecular configuration of the reacting system as conversion proceeds.^64^ The TG model predicted comparatively lower Δ*H* values. The enthalpy change increased from 104.28 kJ mol^−1^ at *α* = 0.1 to 198.59 kJ mol^−1^ at *α* = 0.8, while Δ*G* decreased modestly from 208.90 to 205.81 kJ mol^−1^. In contrast to KAS, DAEM, and OFW, Δ*S* remained negative throughout the conversion range, increasing from −175.49 to −12.11 J mol^−1^ K^−1^. The average Δ*H*, Δ*G*, and Δ*S* values calculated using TG were 152.76 kJ mol^−1^, 207.18 kJ mol^−1^, and −91.27 J mol^−1^ K^−1^. The lower Δ*H* predicted by TG compared with other models demonstrates the influence of the kinetic formulation on the derived thermodynamic parameters.^64^ The positive Δ*H* values obtained from all four models confirm that the thermal decomposition of TXT is endothermic, requiring continuous heat input to overcome the energetic barriers associated with decomposition.^62^ The Δ*G* values remained positive and comparatively stable at approximately 204–209 kJ mol^−1^, despite pronounced changes in Δ*H*. The conversion-dependent changes in Δ*S* were more pronounced, particularly for KAS, DAEM, and OFW, where Δ*S* changed from negative at low conversion to positive at higher conversion.^64^ Collectively, the variation in thermodynamic parameters with conversion and across kinetic models indicates that TXT undergoes multiple thermally activated decomposition stages with distinct energetic requirements rather than a uniform single-step conversion process.

### 3.9. Physicochemical characterization of char

The physicochemical characteristics of TXT-derived char are listed in Table 10. It was observed that TXT-derived char was significantly influenced by pyrolysis temperature (450–800 °C), with progressive carbonisation and structural evolution. Char yield decreased steadily from 51.20 wt% at 450 °C, 45.78 wt% at 600 °C, 44.83 wt% at 700 °C to 43.53 wt% at 800 °C, representing approximately a 15% reduction. This decline is attributed to enhanced thermal decomposition of volatile organic compounds and progressive devolatilization during pyrolysis, resulting in greater mass loss and a higher carbon concentration in the remaining solid matrix.^33^ Among the investigated pyrolysis temperatures (450, 600, 700, and 800 °C), 700 °C was identified as the favourable condition based on a combined qualitative assessment of char yield and physicochemical properties. Although increasing the temperature promoted carbonisation and improved several material properties, further heating to 800 °C reduced char yield and BET surface area. Therefore, 700 °C provided a favourable balance between carbonisation, calorific value, surface development, water-holding capacity, and solid yield. Similar trends have been widely reported for sewage sludge- and biomass-derived char's, in which elevated temperatures accelerate the decomposition of hemicellulosic, cellulosic, and other oxygen-containing constituents.^65^ The moisture content decreased from 3.38 to 2.25 wt%, indicating improved hydrophobicity and thermal stability of the char at higher temperatures. The lower moisture content is advantageous for long-term storage and adsorption applications because it minimises microbial degradation and improves material stability.^24^ Conversely, the ash content increased from 43.0 to 48.48 wt%, reflecting the concentration of inorganic minerals following the removal of volatile organic matter. Textile sludge is inherently rich in mineral constituents, and their enrichment during pyrolysis contributes to the resulting char's alkalinity and catalytic activity.^66^

**Table 10:** Physicochemical characterisation of char obtained at different temperatures

Analysis	TXT-450	TXT-600	TXT-700	TXT-800
Yield (wt%)	51.20 ± 2.15	45.78 ± 1.52	44.83 ± 1.21	43.53 ± 1.11
Moisture content (wt%)	3.38 ± 1.10	2.84 ± 0.82	2.65 ± 0.40	2.25 ± 0.40
Ash content (wt%)	43 ± 0.74	47.95 ± 0.93	48.14 ± 0.72	48.48 ± 0.86
Carbon (wt%)	41.04 ± 0.14	42.92 ± 0.17	44.40 ± 0.16	43.88 ± 0.17
Hydrogen (wt%)	2.12 ± 0.13	0.85 ± 0.11	0.75 ± 0.12	0.43 ± 0.11
Oxygen (wt%)	55.38 ± 0.16	55.27 ± 0.15	53.97 ± 0.13	55.09 ± 0.16
Nitrogen (wt%)	1.44 ± 0.12	0.95 ± 0.11	0.87 ± 0.11	0.59 ± 0.12
Sulphur (wt%)	—	—	—	—
H/C	0.62	0.25	0.20	0.12
O/C	1.01	1.05	0.91	0.97
HHV (MJ kg^−1^)	14.12 ± 1.20	16.19 ± 1.20	18.21 ± 1.18	17.92 ± 1.25
pH	10.27 ± 0.19	11.05 ± 0.17	11.15 ± 0.14	11.21 ± 0.13
Bulk density (kg m^−3^)	724.49 ± 5.84	785.41 ± 5.83	791.87 ± 6.40	799 ± 2.16
BET surface area (m^2^ g^−1^)	8.01 ± 1.20	15.65 ± 1.20	55.23 ± 1.10	30.12 ± 2.10
Water holding capacity (wt%)	45 ± 1.10	60.32 ± 2.11	64.82 ± 2.10	63.04 ± 1.20

The elemental analysis of TXT-derived char confirmed progressive carbonisation. The carbon content increased from 41.04 wt% at 450 °C to a maximum of 44.40 wt% at 700 °C and slightly decreased at 800 °C, while hydrogen and nitrogen contents decreased substantially from 2.12 to 0.43 wt% and 1.44 to 0.59 wt%. The continuous loss of hydrogen and nitrogen indicates dehydration, dehydrogenation, and deamination reactions occurring during pyrolysis, which form a more condensed aromatic carbon structure. Oxygen content remained relatively constant because the high mineral fraction of textile sludge contributes substantially to the calculated elemental composition.^25^ It was found that the elemental properties of char at 800 °C were lower than at 700 °C because prolonged exposure to severe thermal conditions promotes extensive devolatilization, secondary cracking, and mineral transformations at 800 °C. These processes can cause partial carbon gasification and enrichment of inorganic ash, resulting in a slight decrease in carbon content and changes in oxygen composition compared with 450, 600, and 700 °C, despite continued carbonisation. The atomic H/C ratio decreased markedly from 0.62 to 0.12, indicating a significant increase in aromaticity and structural condensation as pyrolysis temperature increased. It was established that chars exhibiting H/C values below 0.3 are generally considered highly carbonised and possess superior chemical stability, making them suitable for long-term environmental applications and carbon sequestration.^67^ Although the O/C ratio fluctuated slightly (0.91–1.05), it remained relatively high because of the substantial inorganic content characteristic of textile sludge-derived char. Nevertheless, the lowest O/C ratio observed at 700 °C suggests partial removal of oxygen-containing functional groups and enhanced aromatic condensation.^67^

The higher heating value (HHV) increased significantly from 14.12 MJ kg^−1^ at 450 °C to 18.21 MJ kg^−1^ at 700 °C, then declined slightly to 17.92 MJ kg^−1^ at 800 °C. The improvement in calorific value is primarily associated with an increase in fixed carbon concentration and a decrease in hydrogen- and oxygen-containing volatile compounds.^68^ The slight reduction at 800 °C may be attributed to excessive devolatilization and the increased proportion of inorganic ash.^68,69^ These results indicate that pyrolysis at approximately 700 °C provides an optimal balance between carbon enrichment and energy content. From Table 10 and it was observed that pH increased from 10.27 to 11.21, indicating increasingly alkaline conditions at higher temperatures. This increase is mainly due to the accumulation of alkali and alkaline-earth metal oxides and carbonates formed during thermal decomposition of inorganic salts. Highly alkaline chars are particularly advantageous for neutralising acidic soils and promoting the adsorption of heavy metals through precipitation and electrostatic interactions.^58^ The bulk density of TXT-derived char increased gradually from 724 to 799 kg m^−3^, suggesting progressive densification of the carbon matrix resulting from shrinkage and structural rearrangement during carbonization. This increase is attributed to enhanced devolatilization, matrix shrinkage, structural rearrangement, and concentration of inorganic mineral matter in the residual char. These changes promote densification of the carbonaceous matrix during progressive thermal treatment. Although higher bulk density can improve handling and transportation efficiency, excessive densification may reduce pore accessibility if accompanied by pore collapse.^33^ The BET surface area of TXT-derived char exhibited the most pronounced improvement, increasing from 8.01 m^2^ g^−1^ at 450 °C to 55.23 m^2^ g^−1^ at 700 °C, followed by a decrease to 30.12 m^2^ g^−1^ at 800 °C. The substantial increase up to 700 °C is attributed to the release of volatile compounds and the development of microporous structures during carbonisation.^68^ Further heating to 800 °C likely caused pore widening, sintering, and partial collapse of the porous framework, resulting in a significant reduction in surface area. The highest surface area obtained at 700 °C suggests that this temperature is optimal for producing char intended for adsorption and catalytic applications. A similar trend was observed for water-holding capacity (WHC), which increased from 45 wt% to 64.82 wt% at 700 °C, then decreased slightly to 63.04 wt% at 800 °C. The enhanced WHC is closely associated with improved pore development and increased internal pore volume, enabling greater water retention.^69^ The slight reduction at the highest temperature supports the hypothesis of pore collapse and structural contraction. The higher WHC is particularly beneficial for soil amendment applications because it improves moisture retention and enhances nutrient availability.^70^ The results demonstrate that pyrolysis temperature strongly influenced the physicochemical properties of textile sludge-derived char. Among the investigated temperatures, 700 °C produced the most favourable combination of carbon content, H/C ratio, HHV, BET surface area, and water-holding capacity, indicating its potential for future investigation in adsorption, environmental, and catalytic applications.

### 3.10. FTIR analysis of char

**Fig. 8:**
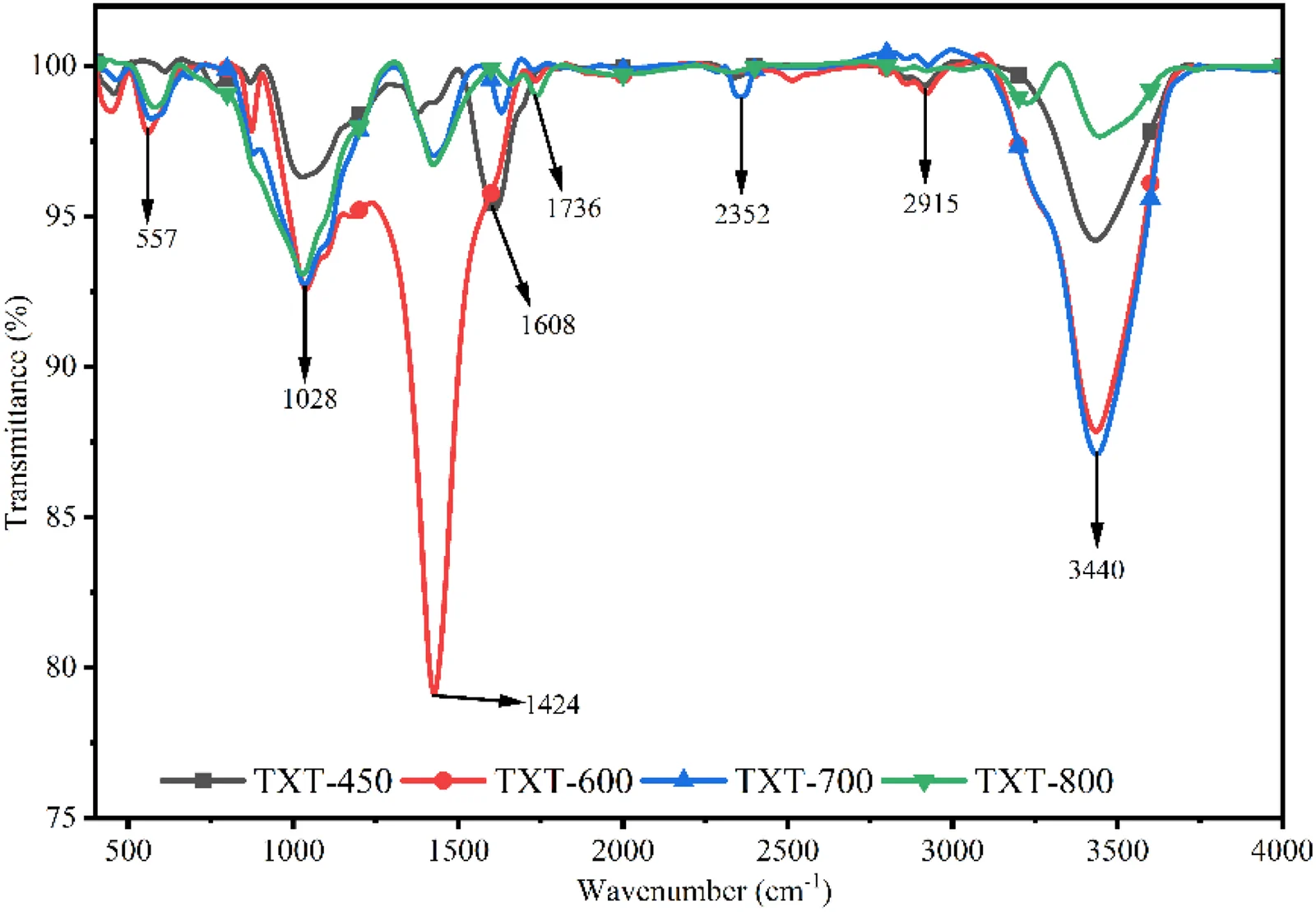
Effect of pyrolysis temperature on the FTIR spectra of textile waste sludge-derived char.

FTIR spectra of char produced at 450, 600, 700, and 800 °C demonstrate a progressive transformation of the surface chemistry with increasing pyrolysis temperature. The plotted spectra of char are presented in Fig. 8. All samples exhibited a broad absorption band around 3440 cm^−1^, corresponding to O–H stretching vibrations of hydroxyl groups and adsorbed moisture.^34^ The intensity of this band decreased markedly at higher temperatures, indicating dehydration and the removal of oxygen-containing functional groups. The absorption band near 2915 cm^−1^, assigned to aliphatic C–H stretching, also weakened with increasing temperature, confirming the decomposition of aliphatic hydrocarbons.^29^ The peaks at 1736 cm^−1^ and 1608 cm^−1^ are attributed to C=O stretching of carbonyl/carboxyl groups and aromatic C=C vibrations.^29^ The carbonyl peak became less pronounced above 600 °C, reflecting decarboxylation and increased aromatic condensation. The strong band at 1424 cm^−1^ observed in TXT-600 is associated with carbonate species or aromatic skeletal vibrations, indicating the formation of stable inorganic carbonates during pyrolysis.^18^ The absorption at 1028 cm^−1^, assigned to Si–O–Si/C–O stretching, persisted in all chars, confirming the retention of silicate minerals and inorganic ash.^34^ The weak band around 557 cm^−1^ corresponds to metal–oxygen vibrations, suggesting the presence of mineral oxides.^18^ Among the prepared chars, TXT-800 exhibited the lowest intensity of oxygen-containing functional groups and the highest degree of aromaticity, indicating superior thermal stability, carbonisation, and structural ordering. In contrast, TXT-450 and TXT-600 retained a higher abundance of oxygenated functional groups, which are beneficial for adsorption through hydrogen bonding and surface complexation. TXT-700 exhibited a significant reduction in oxygen-containing functional groups compared with TXT-450 and TXT-600, as evidenced by the decreased intensity of the O–H (3440 cm^−1^) and aliphatic C–H (2915 cm^−1^) bands. These functional groups were not eliminated, indicating the retention of a moderate number of oxygenated surface sites. TXT-800 is more suitable for applications requiring stable carbon materials (catalyst support and electrode materials), whereas TXT-600 offers a balanced combination of surface functionality and stability, making it the most promising adsorbent for wastewater treatment due to its greater availability of active functional groups.

### 3.11. XRD and SEM analysis of char

**Fig. 9:**
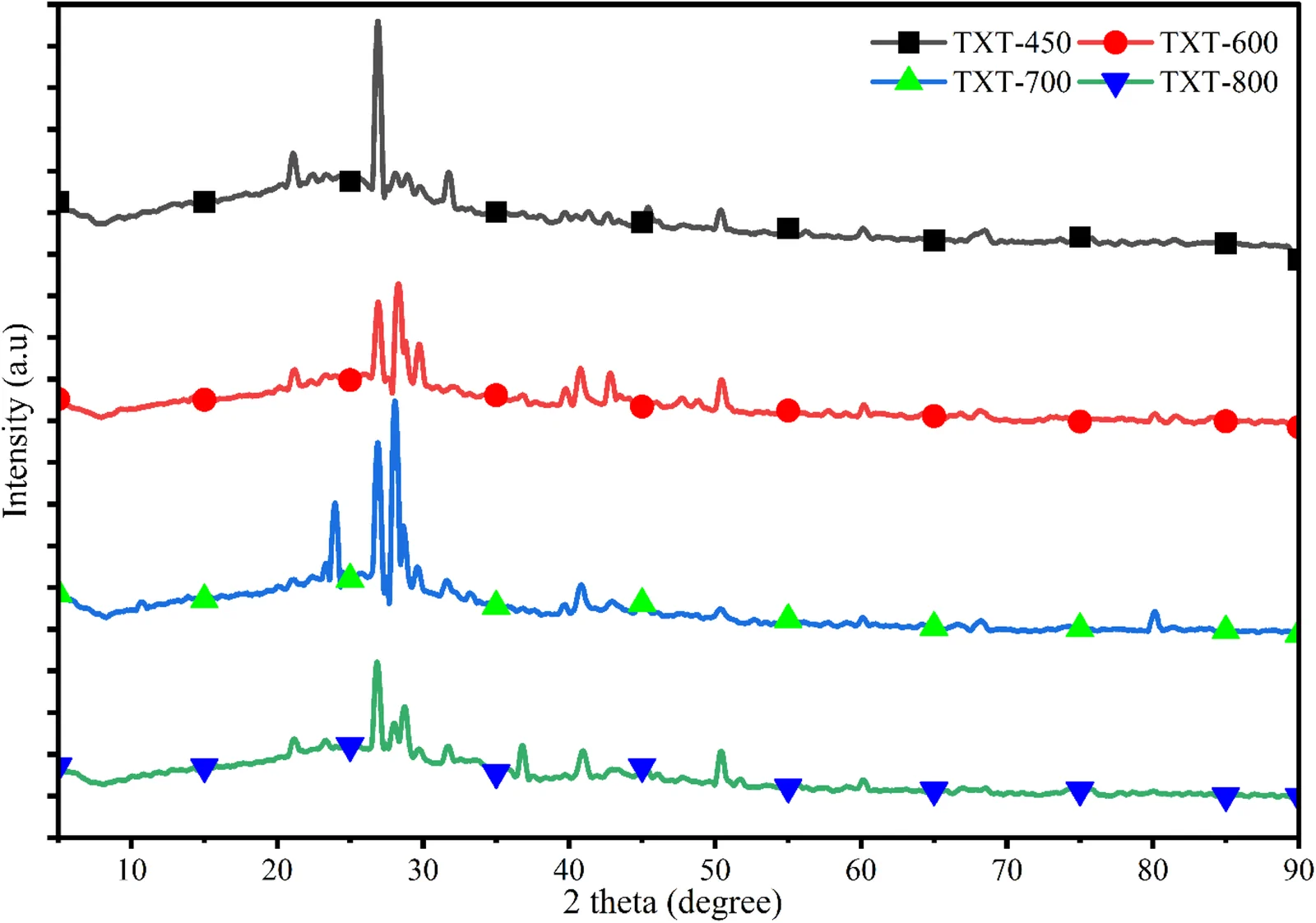
XRD analysis of chars derived at 450, 600, 700 and 800 °C.

The XRD patterns of chars produced at 450, 600, 700, and 800 °C show significant structural and mineralogical transformations with increasing pyrolysis temperature. The XRD analysis of char is listed in Fig. 9. All chars exhibit a broad diffraction halo between 20–30° (2*θ*), characteristic of amorphous carbon, indicating that the carbon matrix remains predominantly non-graphitic after pyrolysis. As the temperature increased from 450 to 800 °C, the diffraction peaks became sharper and more intense, suggesting improved crystallinity due to the decomposition of the organic matrix and the concentration of inorganic minerals in the residual ash. The pronounced reflections at 26.60°, 27.80°, and 29–30° indicate the presence of crystalline silica (quartz), potassium-containing minerals, and calcium-based phases, consistent with the high concentrations of SiO_2_, K_2_O, and CaO identified by ICP-OES. Additional reflections observed between 40 to 55° are attributed to mixed-metal oxides and silicate phases formed during thermal treatment. Among all samples, TXT-700 exhibited the highest peak intensity and the best-defined crystalline phases while retaining a broad amorphous carbon background. This indicates an optimal balance between carbon structural ordering and mineral dispersion, which is desirable for catalytic and adsorption applications, as crystalline mineral phases can provide catalytically active sites, while the amorphous carbon matrix contributes to high adsorption capacity. In contrast, TXT-450 showed broader and less intense peaks, reflecting incomplete carbonisation and lower mineral crystallinity. Although TXT-800 exhibited good crystallinity, the reduction in peak intensity relative to TXT-700 may result from mineral phase transformation, sintering, and partial melting of alkali-rich ash at elevated temperatures. A similar finding was observed in the SEM analysis of chars derived at dynamic temperatures. Excessive thermal treatment can also reduce structural defects and collapse microporous carbon domains, thereby lowering the number of accessible active sites. XRD analysis of chars confirmed that TXT-700 is the optimal char, as it combines a partially ordered carbon framework with well-developed crystalline mineral phases, providing superior physicochemical properties for adsorption and heterogeneous catalysis.

**Fig. 10:**
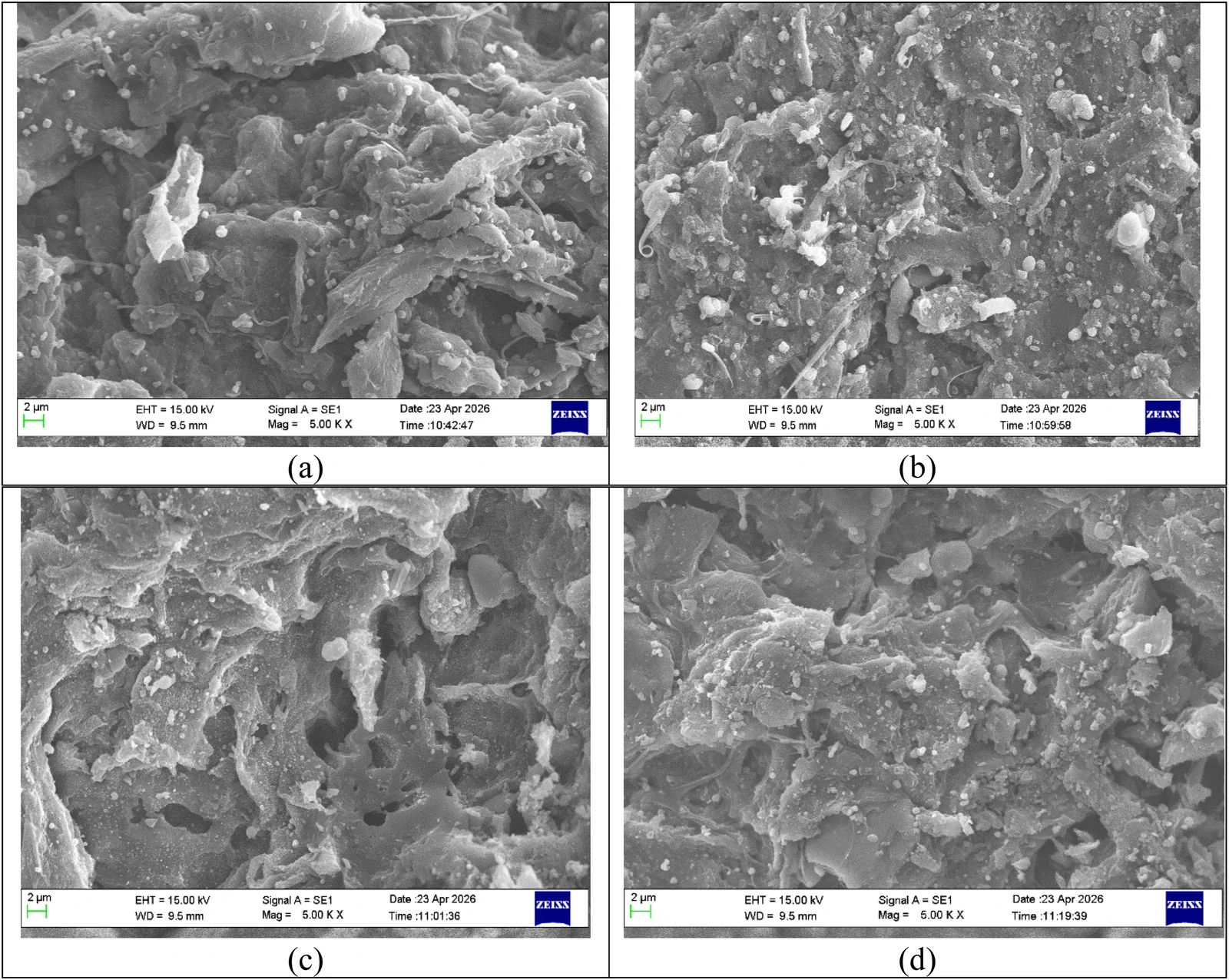
SEM analysis of char (a) 450 °C, (b) 600 °C, (c) 700 °C and (d) 800 °C at 5 KX magnification.

SEM analysis of chars derived at 450, 600, 700, and 800 °C is shown in Fig. 10. SEM micrographs demonstrate that pyrolysis temperature significantly influences the surface morphology and pore development of the textile waste sludge-derived char. It was found that the TXT-450 char surface (Fig. 10(a) appears compact and irregular with limited pore formation. The presence of unconverted organic matter and fused particles indicates incomplete devolatilization, resulting in a relatively low specific surface area. TXT-600 char releases more volatile matter, producing a rougher surface and initiating pore formation (Fig. 10b). Several pores remain partially blocked by residual tar and mineral deposits, indicating that carbonisation is still incomplete.^71^ A well-developed porous structure is observed for TXT-700, with interconnected pores and channels clearly visible due to extensive devolatilization and decomposition of the organic matrix (Fig. 10c). The pore walls remain structurally intact, suggesting an optimal balance between pore generation and structural stability. This morphology is expected to enhance the specific surface area and provide abundant active adsorption sites.^72^ TXT-800 exhibits a more consolidated, interconnected pore and channel structure, with a clear pore structure (Fig. 10d). Although SEM revealed interconnected surface pores at 800 °C, the lower BET surface area indicates that these macro-porous features did not translate into a higher accessible specific surface area, possibly because of pore widening and loss of microporosity. SEM image analysis confirmed that TXT-700 exhibited the most favourable morphology among the investigated chars, combining a highly porous, interconnected structure with good mechanical integrity. TXT-800 is also produced using char of similar quality, but at slightly lower than 700 °C. These properties in char are desirable for adsorption, energy storage and catalytic applications because they facilitate mass transfer, increase the number of accessible active sites, and enhance adsorption efficiency.^72^ Conversely, TXT-450 and TXT-600 are less effective due to incomplete carbonisation and limited pore development and can be used for soil amendment application.^73,74^ Chars produced at high pyrolysis temperatures (>700 °C) are possibly favourable for catalyst support, adsorption, and energy storage applications. These structural changes may result in a high specific surface area, well-developed microporosity, excellent thermal and chemical stability, enhanced electrical conductivity, and a highly aromatic carbon framework, collectively improving catalytic performance, adsorption capacity, and electrochemical properties.^72,75^ Char produced at lower temperatures (400 to 600 °C) is generally most favourable for use as a soil amendment because it provides an optimal balance among nutrient availability, surface functionality, porosity, and long-term stability.^6^ At temperatures below 400 °C, char retains excessive volatile matter and unstable organic compounds, making it more susceptible to microbial degradation and phytotoxicity.^74^

### 3.12. Environmental sustainability and potential risks

The pyrolysis of textile wastewater sludge may offer environmental advantages over disposal-based management by converting the sludge into a carbon-rich solid while reducing the volume of material that requires final disposal. Conventional landfilling does not recover the organic and mineral resources contained in sludge and may also pose long-term concerns regarding contaminant mobility and environmental management. In contrast, pyrolysis enables partial recovery of carbon and inorganic constituents in the solid product and therefore has potential relevance to circular-economy strategies. However, the present study did not directly quantify greenhouse gas emissions or compare the environmental footprint of pyrolysis with that of landfilling; therefore, the magnitude of any greenhouse gas reduction cannot be established from the present results. A process-level life-cycle assessment would be required to quantify the net environmental benefit. The relatively high carbon content of the chars, particularly at higher pyrolysis temperatures, indicates increased carbonisation and retention of carbon in the solid phase. Such carbon retention may provide an opportunity for longer-term carbon storage when the resulting char is appropriately managed. Nevertheless, carbon sequestration potential depends on char stability, energy requirements for production, carbon losses during pyrolysis, and the eventual application and fate of the char. The present carbon-content measurements should be interpreted as an indication of carbon retention rather than direct evidence of net carbon sequestration. The environmental application of textile wastewater sludge-derived char requires careful consideration of inorganic contaminants. The feedstock contained substantial mineral constituents, including K_2_O, SiO_2_, P_2_O_5_, CaO and Na_2_O, indicating significant mineral enrichment in the resulting char. During pyrolysis, non-volatile inorganic constituents may become concentrated in the solid phase, and the resulting char may therefore require assessment of total heavy-metal concentrations, chemical speciation, and leaching behaviour before environmental application. Although such analyses were beyond the scope of the present study, they are essential for determining the environmental safety of textile wastewater sludge-derived char. Future work should therefore include standardised leaching tests and assessment of metal mobility under relevant environmental conditions.

## 4 Conclusions and future research scope

This study provides an integrated assessment of the thermal conversion behaviour and temperature-dependent char properties of textile wastewater sludge. The multi-model kinetic analysis demonstrated strongly conversion-dependent degradation, with average apparent activation energies of 213.03, 208.81, 218.61, 157.72, and 170.14 kJ mol^−1^ obtained using KAS, OFW, DAEM, Tang, and VZ methods, respectively. The consistent increase in activation energy with conversion confirms the multistep nature of sludge decomposition rather than a single reaction pathway. Criado master-plot analysis further indicated a transition from predominantly diffusion-related behaviour at lower conversion to reaction-order and nucleation-related contributions at higher conversion, reflecting the progressive depletion of readily degradable fractions and the formation of a more resistant carbonaceous matrix. Pyrolysis temperature strongly influenced char characteristics. Char yield decreased from 51.20 to 43.53 wt% as temperature increased, while carbonisation and several material properties improved. Among the investigated temperatures, 700 °C provided the most favourable balance of solid yield and physicochemical characteristics, including enhanced surface development, calorific value, and water-holding capacity. These characteristics suggest potential applicability in adsorption, catalysis, soil amendment, and other environmental applications. However, the present study did not include application-specific performance testing, and these potential applications should be considered preliminary. Future studies should evaluate the adsorption, catalytic, electrochemical, and agronomic performance of the produced char to validate its practical suitability. This integrated kinetic-material assessment provides new insight into the relationship between sludge conversion behaviour and char evolution.

Future studies should focus on scaling up textile waste sludge pyrolysis from laboratory-scale fixed-bed systems to pilot and continuous reactors to evaluate process stability, energy efficiency, and techno-economic feasibility. The influence of operating parameters such as heating rate, residence time, particle size, nitrogen flow rate, and reactor configuration should be systematically optimised to maximise char quality and energy recovery. Since the ash contains substantial alkali and mineral components, detailed investigations of heavy-metal speciation, leaching behaviour, ash transformation, and long-term environmental stability are required before large-scale application of char. The excellent porosity and surface characteristics of TXT-700 indicate its favourable potential for adsorption and catalytic applications. Its performance should be experimentally evaluated for the removal of dyes, heavy metals, pharmaceuticals, and other emerging contaminants from wastewater. Surface modification and activation could further improve adsorption performance. Life-cycle assessment, techno-economic analysis, regeneration studies, and carbon-sequestration evaluation are recommended to establish the environmental and commercial sustainability of textile sludge-derived char.

## Author contributions

Gaurav Singh: data curation, investigation, visualisation, writing – original draft, Neeraj Kumar: visualisation, editing original draft, and supervision, Ranjeet Kumar Mishra: conceptualisation, data curation, methodology, investigation, visualisation, editing original draft, and supervision.

## Conflicts of interest

The author declares that they have no known competing financial interests or personal relationships that could have appeared to influence the study reported herein.

## Abbreviations

KAS: Kissinger–Akahira–Sunose
OFW: Ozawa–Flynn–Wall
DAEM: Distributed activation energy model
TG: Tang method
VZ: Vyazovkin method
TGA: Thermogravimetric analysis
Δ*H*: Enthalpy change
Δ*G*: Gibbs free energy change
Δ*S*: Entropy change
MC: Moisture content
VM: Volatile matter
FC: Fixed carbon
HHV: Higher heating value
*A*
: Frequency factor
*E*_a_
: Apparent activation energy
WHC: Water holding capacity
ETPS: Effluent treatment plant sludge

## Data Availability

The datasets generated and/or analysed during the current study are not publicly available but are available from the corresponding author on reasonable request.
